# Computer vision for pattern detection in chromosome contact maps

**DOI:** 10.1038/s41467-020-19562-7

**Published:** 2020-11-16

**Authors:** Cyril Matthey-Doret, Lyam Baudry, Axel Breuer, Rémi Montagne, Nadège Guiglielmoni, Vittore Scolari, Etienne Jean, Arnaud Campeas, Philippe Henri Chanut, Edgar Oriol, Adrien Méot, Laurent Politis, Antoine Vigouroux, Pierrick Moreau, Romain Koszul, Axel Cournac

**Affiliations:** 1 Institut Pasteur, Unité Régulation Spatiale des Génomes, CNRS, UMR 3525, C3BI USR 3756, Paris, France; 2 Sorbonne Université, Collège Doctoral, F-75005 Paris, France; 3 ENGIE, Global Energy Management, Paris, France; 4 Institut Pasteur, Synthetic Biology Group, Paris, France

**Keywords:** Bioinformatics, Nuclear organization, Data processing, Genome informatics

## Abstract

Chromosomes of all species studied so far display a variety of higher-order organisational features, such as self-interacting domains or loops. These structures, which are often associated to biological functions, form distinct, visible patterns on genome-wide contact maps generated by chromosome conformation capture approaches such as Hi-C. Here we present Chromosight, an algorithm inspired from computer vision that can detect patterns in contact maps. Chromosight has greater sensitivity than existing methods on synthetic simulated data, while being faster and applicable to any type of genomes, including bacteria, viruses, yeasts and mammals. Our method does not require any prior training dataset and works well with default parameters on data generated with various protocols.

## Abstract (web summary)

Chromatin loops bridging distant loci within chromosomes can be detected by a variety of techniques such as Hi-C. Here the authors present Chromosight, an algorithm applied on mammalian, bacterial, viral and yeast genomes, able to detect various types of pattern in chromosome contact maps, including chromosomal loops.

## Introduction

**Fig. 1:**
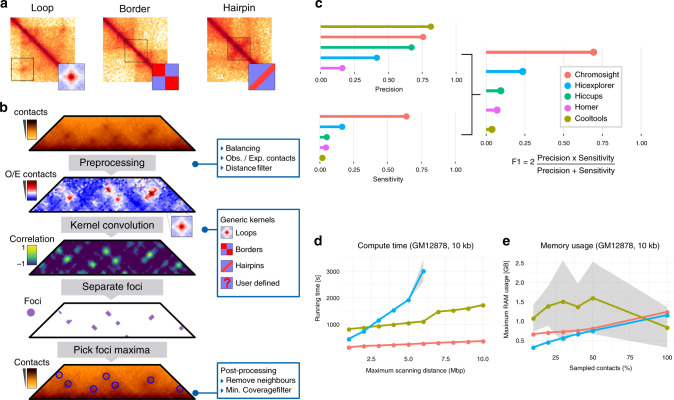
Chromosight workflow and benchmark. **a** Examples of distinct patterns visible on contact maps (loop, border and hairpin) and the corresponding chromosight kernels. **b** Matrix preprocessing involves normalisation balancing followed by the computation of observed/expected contacts. Only contacts between bins separated by a user-defined maximum distance are considered. The preprocessed matrix is then convolved with a kernel representing the pattern of interest. For each pixel of the matrix, a Pearson correlation coefficient is computed between the kernel and the surrounding window. A threshold is applied on the coefficients and a connected component labelling algorithm is used to separate groups of pixels (i.e. foci) with high correlation values. For each focus, the coordinates with the highest correlation value are used as the pattern coordinates. Coordinates located in poorly covered regions are discarded. **c** Comparison of Chromosight with different loop callers. Top: F1 score, Precision and Sensitivity scores assessed on labelled synthetic Hi–C data. Higher is better. **d** Run-time. **e** Memory usage according to maximum scanning distance and the amount of subsampled contact events, respectively. Means and standard deviations (grey areas) are plotted.

Proximity ligation derivatives of the chromosome conformation capture (3C) technique^1^ such as Hi–C^2^ or ChIA-PET^3^ determine the average contact frequencies between DNA segments within a genome, computed over hundreds of thousands of cells.These approaches have unveiled a wide variety of chromatin 3D structures in a broad range of organisms. For instance, in all species studied so far, sub-division of chromosomes into self-interacting domains associated with various functions have been observed^4,5^ (Fig. 1a). In addition, chromatin loops bridging distant loci within a chromosome (from a few kb to a Mb) are also commonly detected by Hi–C, such as during mammalian interphase^6^ or yeast mitotic metaphase^7–9^. Other spatial structures are more peculiar, and sometimes specific to some organisms. For instance, the contact maps of most bacteria display a secondary diagonal perpendicular to the main one^10–12^, reflecting the bridging of chromosome replichores (i.e. arms) by the structural maintenance of chromosome complex (SMC) condensin^10^, a ring-shaped molecular motor able to entrap and travel along DNA molecules^13^. Smaller straight, or loosely bent, secondary diagonals, also perpendicular to the main diagonal, can also be observed in some maps, reflecting potentially long DNA hairpins or dynamic sliding asymmetrical contacts (Fig. 1a). Such “hairpin-like” configuration is for instance observed near the origin of replication of the *Bacillus subtilis* genome, were it was originally described as a “bow shaped” structure^10^. The formation of these different structures can vary depending on the stage of the cell cycle,^7,10,14^, the state of cell differentiation^15^ or viral infection^16^. Different molecular mechanisms have been proposed to explain the patterns visible on the contact maps, and for a similar pattern, these mechanisms or their regulation can differ. Although detailing these mechanisms is beyond the scope of the present work, one can note that in mammals the CCCTC-binding factor (CTCF) protein is enriched at loop anchors (i.e. the regions bridged together). It has been proposed that CTCF acts as a roadblock to the SMC molecular motor cohesin, which travels along chromatin. Cohesins promote the formation of chromatin loops, potentially through a loop extrusion mechanisms in which two chromatin filaments are extruded through the cohesin ring^17^). When cohesin encounters a roadblock along one of the filament, chromatin displacement stops in this direction. As a consequence, two roadblocks at two distant loci will stop cohesin progression along both filaments, resulting in a stabilised loop. Such stable loops are then visible in bulk genomics techniques such as Hi–C (for more insights on the putative mechanisms, see for instance^17,18^). Other patterns such as the perpendicular “hairpin” can be explained by alternative scenarios, for instance where cohesin is continuously loaded at a discrete position along the chromatin while being unloaded before hitting a roadblock. A single roadblock combined with continuous cohesin loading in an adjacent locus could result in a bent, bow-shaped pattern, as proposed in^10,19,20^. A large body of work, exploiting genetics and chromosome engineering approaches, aims at characterising the regulation and the functional relationships of these 3D features with DNA processes such as repair, gene expression or segregation. Although most structural features can be identified by eye on the contact maps, automated detection is essential to quantify and facilitate the biological and physical interpretation of the data generated through these experiments. While border detection can be achieved quite efficiently using different methods (segmentation, break-point detection, etc; ref. ^21^), the calling of loops, as well as other more peculiar features such as “hairpin-like” signals, remains challenging.

Most tools aiming at detecting DNA loops in contact maps rely on statistical approaches and search for pixel regions enriched in contact counts, such as Cloops^22^, HiCCUPS^23^, HiCExplorer^24^, diffHic^25^, FitHiC2^26^, HOMER^27^. These programs can be computationally intensive and take several hours of computation for standard human Hi–C datasets (reviewed in ref. ^22^), or require specialised hardware such as GPU (HiCCUPS). In addition, most if not all of them were developed from, and for, human data. As a consequence, they suffer from a lack of sensitivity and fail to detect biologically relevant structures not only in non-model organisms but also in popular species with compact genomes such as budding yeast (*Saccharomyces cerevisiae*) or bacteria where the scales of the structures are considerably smaller than in mammalian genomes. Here we present *Chromosight*, an algorithm that, when applied on mammalian, bacterial, viral and yeast genome-wide contact maps, quickly and efficiently detects and/or quantifies any type of pattern, with a specific focus on chromosomal loops. Different species were chosen to reflect the diversity of genome-wide contact maps observed in living organisms. For instance, loop contact patterns have been observed in these four clades, but with very different scales and visibility. In human (genome size: ~3 Gb), interphase chromosomes display loops bridging chromatin loci separated by  ~20 kb to 20 Mb. The structures are reflected by well-defined, discrete dots in the contact maps, away from the main diagonal. In contrast, the mitotic chromosomes of *S. cerevisiae* and fission yeast *Schizosaccharomyces pombe* (genome sizes: ~12 Mb) organise into arrays of loops spanning ~5–50 kb, i.e. much smaller than the loops observed along mammalian interphase chromosomes^7–9^. Because of their proximity to the main diagonal in standard Hi–C experiments, the signal generated by those loops is more difficult to call. Loops have been observed in bacteria as well. For instance, in *B. subtilis* (genome size: 4.1 Mb), a few weak, discrete loop signals were observed but never directly quantified^10^. In addition to loops, self-interacting domains have also been described in these different species, that differ in size and nature. For instance, topologically associating domains^4,28^ have a mean size of 1 Mb (from 200 kb to 6 Mb) in human and mice, compared to the small, chromosome interacting domains (CID) of bacteria that range in size between a few dozens to a couple hundreds kb^10,29,30^. Besides this limitation, most programs are limited to domain or loop calling and remain unable to call de novo different contact patterns such as DNA hairpins or the asymmetric patterns seen in species such as *B. subtilis*^10^.

## Results

### Presentation and benchmark of Chromosight

Chromosight takes a single, whole-genome contact map in sparse and compressed format as an input. It applies a balancing normalization procedure^31^ to attenuate experimental biases. A detrending procedure, to remove distance-dependent contact decay due to polymeric behaviour, is then applied, which consists in dividing each pixel by its expected value under the polymer behaviour (Fig. 1b). A template (kernel) representing a 3D structure of interest (e.g. a loop, a boundary,...) is fed to the program and sought for in the image of the contact map through two steps (Fig. 1b). First, the map is subdivided into sub-images correlated to the template; then, the sub-images with the highest correlation values are labelled as template representations (i.e. potential matches, see Methods). Correlation coefficients are computed by convolving the template over the contact map. To reduce computation time, the template can be approximated using truncated singular value decomposition (tSVD) (Supplementary Note 1^32^). To identify the regions with high correlation values (i.e. correlation foci), Chromosight uses Connected Component Labelling (CCL). Finally, the maximum within each correlation focus is extracted and its coordinates in the contact map determined.

We decided to benchmark Chromosight against 4 existing programs by running them in loop-calling mode on synthetic Hi–C data mimicking mitotic chromosomes of *S. cerevisiae* (“Methods” and Supplementary Fig. 1). Whereas Chromosight displays a precision (i.e. proportion of true positives among detected patterns) comparable to the other programs, its sensitivity (i.e. proportion of relevant patterns detected) is more than threefold higher (~70%) compared to the second-best program Hicexplorer (~20%) (Fig. 1c). As a result, Chromosight’s F1 score, a metric that considers both precision and sensitivity, is also threefold higher, reflecting the effectiveness of the program at detecting more significant loops in this synthetic case study (Supplementary Fig. 2a). To further benchmark the program’s performance, we ran the three best CPU-based programs (Cooltools, Hicexplorer, Chromosight) on high resolution (10 kb), human genome-wide experimental contact maps. Chromosight outperforms existing methods regarding computing time (Fig. 1d), without straining RAM (Fig. 1e). For instance, on a single CPU core, it detects loops at maximum distance of 5 Mb within ~5 min compared to ~17 and 30 min for Cooltools and Hicexplorer, respectively.

To get a sense of the differences between the softwares when applied to experimental human contact maps, we compared them with default parameters on Hi–C data generated from GM12878 cell lines^33^. Compared to Chromosight, we first noticed that other programs missed multiple loops which were clearly visible on the maps (e.g. Supplementary Fig. 3a). For instance, Chromosight found 85% of the loops detected by Cooltools, the software with the highest precision in our benchmark, while overall identifying a much larger number of loops (37,955 vs. 6264, respectively) (Supplementary Fig. 3c). We then measured the proportion of loops with both anchors overlapping CTCF peaks identified from ChIP-seq^34^. Almost all (~95%) loops detected by Hiccups and Cooltools, the most conservative programs, co-localize with CTCF enriched sites, compared to ~64% for the loops detected by Chromosight and Hicexplorer (Supplementary Fig. 3b). Chromosight (and Hicexplorer) indeed detects multiple weaker loops, visible on the maps and arranged in grid-like patterns, but often with only one anchor falling into a well-defined CTCF enriched site. Some of these weaker loops’ anchors may be less enriched in CTCF, which would cause ChIP-seq peak calling algorithms to discard them because of parameters such as intensity thresholds, or minimum inter-peak distances. This means that more sensitive loop callers could result in lower CTCF peak overlap, not because of inaccurate detection, but rather because of the CTCF peaks cutoffs. On the other hand, less sensitive loop callers would call the strongest loops associated with the strongest CTCF peaks. We can also not exclude that a portion of the less intense loops called by Chromosight are linked to different protein complexes or mechanisms. More investigations will further dissect the nature of these loops.

### Detection and quantification of loops in a compact genome

**Fig. 2:**
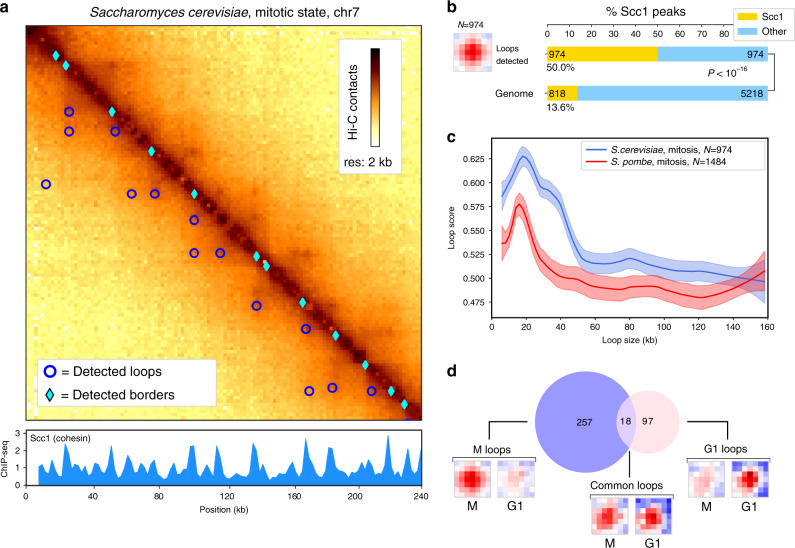
Applications on yeast genomes. **a** Zoom-in of the contact map of chromosome 5 of *S. cerevisiae* with synchronised ChIP-Seq signal of Scc1 protein (cohesin) at 2 kb resolution with detected loops and border patterns,^8^. The darker, the more contacts. **b** Pileup plots of windows centered on detected loops with the number of detections. Barplots of the proportion of Scc1 peaks for anchors of detected loops and associated *p*-value (Fisher test, two-sided). **c** Loop spectrum showing scores in function of the loop size in *S. cerevisiae* (974 loops) and *S. pombe* (1484 loops). Curves represent lowess-smoothed data for easier interpretation with 95% confidence intervals. **d** Number of loops detected only in G1 phase, M phase, or in both. For each category, the pileup of each set of coordinates is shown for both G1 and M conditions (mitotic data^8^ subsampled from 44M to 5.8M contacts for comparison with G1^7^).

Hi–C contact maps of budding and fission yeast chromosomes generated from synchronised cells during meiosis^35^ and mitosis^7–9^ display arrays of chromatin loops. Recent work further showed that *S. cerevisiae* mitotic loops are mediated and regulated by the SMC complex cohesin^7,8^. Chromosight loop calling on data from ref. ^8^ identified 974 loops along *S. cerevisiae* mitotic chromosomes (Fig. 2a). An enrichment analysis shows that half (50%) of the anchors of those mitotic loops consist in loci enriched in the cohesin subunit Scc1 (Fig. 2b), (*P*  < 10^−16^). The loop signal spectrum in mitosis shows the most stable loops are ~20 kb long (Fig. 2c). This size is also found in the *S. pombe* yeast, which has longer chromosomes.

On the other hand, loop calling on contact maps generated from cells in G1, where cohesin does not stably binds to chromosomes, yielded only 115 loops (Fig. 2d and Supplementary Fig. 4a). Interestingly, this pool of loops appears different from the group of loops detected during mitosis suggesting that cohesin independent processes act on chromosomal loop formation in yeast (Fig. 2d and Supplementary Fig. 4a). Notably, loop anchors were enriched in highly expressed genes (HEG) (Supplementary Fig. 4a).

To validate the biological relevancy of the loops detected by Chromosight during mitosis, we further analysed their dependency and association to cohesin using the quantification mode implemented in the program (Methods and Supplementary Fig. 5a). This mode allows to precisely compute the correlation scores on a set of input coordinates with a generic kernel. We computed the “loop spectrum” (Loop score versus size) for pairs of cohesin ChIP-seq peaks separated by increasing genomic distances. A characteristic size of 20 kb was clearly visible on the spectrum during mitosis, whereas the spectrum in G1 appeared flat (Supplementary Fig. 5b). This analysis highlights the role of cohesin in mediating regular loop structures during mitosis and shows how Chromosight can be used to precisely quantify spatial patterns like chromosome loops.

To test the ability of Chromosight to detect loops in a genetically disturbed context, they were called on contact data of a mutant depleted for the SMC holocomplex member Pds5 (Precocious Dissociation of Sisters)^7^. This protein regulates cohesin loop formation through two independent pathways^7^, and its depletion leads to the formation of loops over longer distances than in wild-type yeast. One anchor of loops in Pds5 depleted cells appeared to be the centromeres, as suggested by visual inspection of the maps^7^. However, loop patterns are shadowed by a strong boundary signal appearing at the centromeres, which makes their visual identification challenging. Loop calling using Chromosight confirmed this observation, as the anchors of the loops called were strongly enriched at centromeric regions (Supplementary Fig. 4b, *P*  < 10^−16^)). This analysis shows that Chromosight is able to robustly quantify global reorganisation of genome architecture.

Finally, we called domain boundaries (Fig. 1a, border kernel) on the G1 maps, identifying 473 instances of boundaries mostly associated with HEG as well (Supplementary Fig. 4b).

### Exploration of various genomes and patterns

**Fig. 3:**
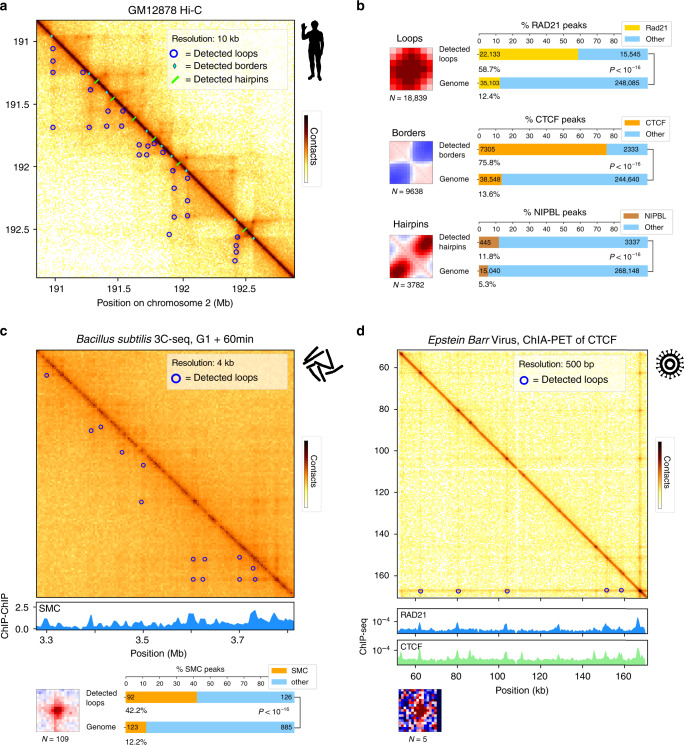
Applications to various genomes. **a** Zoom-in of contact map for chromosome 2 of *Homo sapiens* at 10 kb resolution^36^ with Chromosight detection of loop, border and hairpin patterns. The darker, the more contacts. **b** Left: pileup plots of windows centered on detected loops, borders and hairpins with the number of detections. Right: bar plots showing proportion in Rad21 peaks for detected loops, proportion in CTCF peaks for detected borders and proportion of NIPBL peaks for detected hairpins and associated *p*-value (Fisher test, two-sided). **c** Detection of loops in the *B. subtilis* genome. Subset of the *B. subtilis* genome-wide contact map near the replication origin. The darker, the more contacts. Loops are called with Chromosight and annotated with blue circles. Under the contact map the ChIP-chip signal deposition of *B. subtilis* SMC is plotted^10^.  The pileup plot of the detected loops, and a bar plot showing enrichment of SMC in the anchors of the detected loops (Fisher test, two-sided), are indicated underneath. **d** Contact map of the Epstein Barr virus genome^38^. Called loops using Chromosight are indicated with blue circles. The ChIP-seq deposition signal of Rad21 and CTCF is plotted under the map. Associated pileup plot of the detections is indicated underneath.

To further test the versatility of Chromosight, we called all three kernels described in Fig. 1a, i.e. loops, borders and hairpins (Supplementary Fig. 6) in Hi–C contact maps of human lymphoblastoids (GM12878)^36^ (Fig. 3a).

With default parameters, Chromosight identified 18,839 loops (compared to ≃10,000 detected in ref. ^6^) whose anchors fall mostly (~ 58%, *P* < 10^−16^) into loci enriched in cohesin subunit Rad21 (Fig. 3b). Decreasing the detection threshold (Pearson coefficient parameter) allows to detect lower intensity but relevant patterns (Supplementary Fig. 7a). The program also identified 9638 borders, ~75% of which coincide with CTCF binding sites, compared to ~14% expected (*P* < 10^−16^). In human, TADs are known to be delimited by CTCF-enriched sites, suggesting that Chromosight does indeed correctly identify boundaries involved in TADs delimitation. Finally, Chromosight detected 3,782 hairpin-like structures (Fig. 3b), a pattern not systematically sought for in Hi–C maps. The chromosome coordinates for this pattern appeared enriched in cohesin loading factor NIPBL (2 fold effect, *P* < 10^−16^), suggesting that these hairpin-like structures could be interpreted as cohesin loading points (Supplementary Fig. 6). To test for a role of cohesin and NIPBL in generating these patterns, we quantified loops and hairpins on contact maps generated from cells depleted either in cohesin or NIPBL. Both conditions were associated with a disappearance of the detected patterns (Supplementary Fig. 8), further supporting their formation hypothesis. Finally, we called loops de novo along the genomes of various animals from the DNA Zoo project^37^, showing that stable loops of ≃100–150 kb are a conserved feature of animal genomes (Supplementary Fig. 9).

The loop detection efficiency was also tested using noisier, compact genomic contact maps. We applied it on the 3C-seq data generated from bacterium *B. subtilis*^10^. Chromosight identified 109 loops distributed throughout the chromosome (Fig. 3c). Annotation of loop anchor positions showed a strong enrichment with the bacteria Smc-ScpAB condensin complexes (Fig. 3c). Some of these loops were surprisingly large, bridging loci separated by more than 100 kb (Supplementary Fig. 10) (for a genome size of 4.1 Mb). Several of these large loops may correspond to the bridging of replichores at positions symmetric with respect to the origin of replication (Supplementary Fig. 10). This is in agreement with^10^ which showed how SMC condensin SMC-ScpAB complexes loaded at sites adjacent to the origin of replication of the chromosome tether the left and right chromosome arms together while traveling from the origin to the terminus.

Finally, we used Chromosight to detect loops on contact data generated using pair-end tag sequencing (ChIA-PET)^38^, which captures contacts between DNA segments associated to a protein of interest. We used ChIA-PET data for CTCF from human lymphoblastoids^38^ binned at a very high resolution (500 bp). Lymphoblastoids are immortalised B lymphocytes, they contain episomes of the Epstein Barr Virus (EBV), a DNA virus that is approximately 172 kb in size and is involved in the development of certain tumours^39^. Surprisingly, Chromosight detected several loops (5) inside the genome of the Epstein Barr virus^38^. These loops, of a few dozen kb in size, coincide with the position of the cohesin (Rad21) and CTCF binding sites present along the viral genome (Fig. 3d). Such interactions have been suggested from 3C qPCR data^40^. Automatic detection now unambiguously supports a specific viral chromosome structure that could impact the transcriptional regulation and metabolism of the virus^40^.

### Application to different proximity ligation protocols

**Fig. 4:**
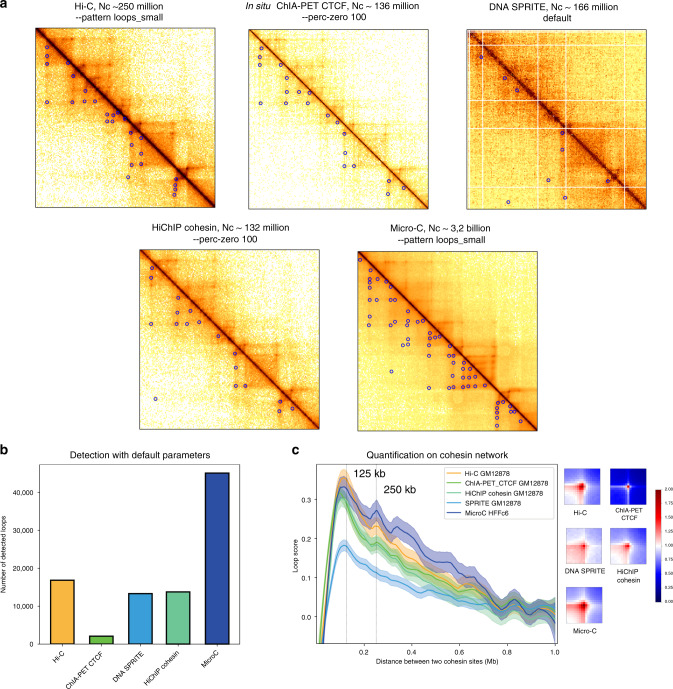
Analyses with data from alternative contact technologies. **a** Magnification of *Homo sapiens* chromosome 2 contact maps generated with five different experimental methods (around STAT1 gene; bin:10 kb): Hi–C^36^, In situ ChiA-PET of CTCF^42^, DNA SPRITE^43^, HiChIP of cohesin^44^, Micro-C^45^. All cells are cycling GM12878 cell types except for Micro-C (hESC). Blue circles: loops detected using Chromosight. The corresponding number of reads in each of the genome-wide map is indicated above the panels. The parameter (if any) notified to Chromosight is also indicated above each map. **b** Number of loops detected using Chromosight with default parameters for the five datasets. **c** Left: loop spectrum computed using Chromosight in quantify mode on pairs of cohesin peaks for the five datasets (Methods). Curves represent lowess-smoothed data with 95% confidence intervals. Right: associated pileup plots of the quantified positions for the five different experimental methods.

Besides Hi–C, Chromosight can be applied on contact data generated with alternative protocols developed to explore various aspect of chromosomal organisation (Fig. 4a). We retrieved publicly available datasets from asynchronous human cells spanning a range of techniques (i.e. ChIA-PET, DNA SPRITE, HiChIP and Micro-C) from the 4D Nucleome Data Portal^41^, and applied loops detection in the resulting contact maps. In situ ChIA-PET^42^ quantifies the contact network mediated by a specific protein of interest thanks to the addition of an immunoprecipitation step. Chromosight required adjustment of a single parameter to produce visually satisfying loop calling in in situ ChIA-PET data. We then performed loop detection on DNA Split-Pool Recognition of Interactions by Tag Extension (SPRITE) data^43^. This approach requires cross-linking and fragmentation of chromatin but does not use ligation. Instead, it splits the content into 96-well plates with barcode molecules in each well. The barcode signature allows clustering of complexes that were originally part of a higher-order chromatin structure in the nucleus. Chromosight was able to detect patterns that visually correspond to loops, although the noise present in this original proof-of-principle dataset made detection challenging. We then analysed HiChIP data^44^, a protocol similar to ChIA-PET but with a better signal-to-noise ratio, and that requires a lower amount of input DNA. The results of loop calling on HiChIP matrices were very close to those from Hi–C (Fig. 4a). Finally, loops were called on the Micro-C data recently generated from human embryonic stem cells (hESC)^45^. Micro-C uses MNase digestion and a dual crosslink procedure, which allows a contact resolution down to the nucleosome scale. This approach resulted in the highest number of loops (~45,000 Fig. 4b); a visual inspection confirmed that most of them appeared relevant. The number of detected loops in each protocol is directly dependent on the coverage, but these analyses show that Chromosight can conveniently be used for the analysis of data generated through various proximity ligation protocols with minimal, if any, tuning.

In parallel to the loop calling mode, we also used Chromosight in its quantify mode to measure the loop signal between pairs of cohesin peaks as a function of their genomic distance for the different protocols in asynchronous human cells (Fig. 4c). The resulting spectra were quite similar, with loop scores peaking around 120 kb for each protocol. Surprisingly, a secondary peak was also clearly visible at 250 kb, corresponding to about twice the fundamental frequency. This peak was clearest with the Micro-C data. These peaks were absent from dataset generated directly on mitotic condensed chromosomes (*T* = 0 from ref. ^46^), but using the same ChIP-seq dataset (Supplementary Fig. 8c). The median distance between cohesin peaks called from ChIP-seq was 468 kb, suggesting that this parameter didn’t introduce a bias accounting in the 120 kb. This double peak in the distribution of cohesin contacts as a function of their genomic distance in interphase cells remains to be validated independently, and its signification characterised.

### Point and click mode

In addition to the kernels presented here (loops, borders, hairpins), visual inspection of the contact maps may inspire scientists to seek for new patterns of interest for quantitative analysis. We have therefore included a “point and click” mode that allows easy manual inspection of Hi–C contact maps to select patterns identified by users. The user clicks on positions corresponding to patterns of interests. For each position, a window will be drawn by the program. A new kernel is then automatically generated by summing all windows and applying a Gaussian filter to attenuate the fluctuations resulting from the small number of selected positions. This kernel can then be used in the other modes of Chromosight (detection, quantification) for further analyses.

We illustrate this functionality to investigate the pattern of centromere-centromere interactions in yeast. Yeasts contact maps are scattered with cross-shaped dots corresponding to inter-chromosomal contacts between peri-centromeric positions. This cross-shaped pattern is characteristic of the Rabl configuration of those genomes, where all centromeres are maintained in the vicinity of each other at the level of the microtubule organising center^47,48^. As a result, peri-centromeric regions collide with each other more frequently than with the rest of the genome, resulting in a distinct trans pattern. In budding yeast, the 16 centromeres result in 120 discrete, inter-chromosomal cross-shaped dots. We selected (by double-clicking) 15 patterns of these *S. cerevisiae* centromere contacts. The resulting kernel was then used to perform the detection of similar structures in the genome contact map of another yeast species, *Candida albicans*, a diploid opportunistic pathogen which contains 8 pairs of chromosomes (resolution: 5 kb, ref. ^49^).

**Fig. 5:**
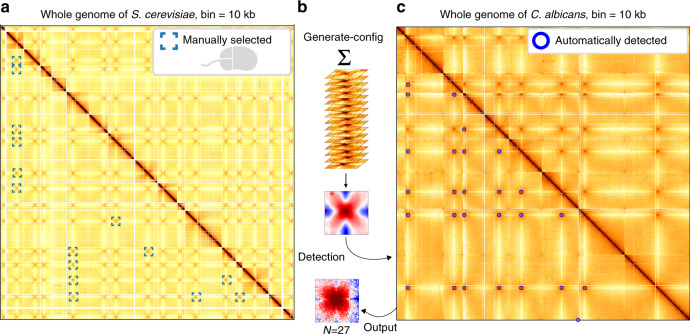
Point and click mode. **a** Whole-genome contact map of *S. cerevisiae*^8^ with 15 inter-centromere patterns that were selected by hand. Darker means more contacts. **b** Chromosight generates a new kernel by summing all the selected patterns and applying a Gaussian filter. **c** Chromosight detection of the inter-centromeres patterns in the whole-genome contact map of *C. albicans*^49^ with the resulting pileup plot of the 27 detections.

Using the kernel generated de novo from the *S. cerevisiae* contact map, Chromosight automatically detected 26 out of the 28 inter-centromeric patterns of *C. albicans*, along with one false positive (most likely a genome misassembly, located at the edge of the map) (Fig. 5). These positions are nevertheless sufficient to point at centromere positions, and can for instance then be used to characterise their genomic coordinates^47^.

Note that, although subtelomeric regions in yeast tend to cluster in yeast nuclei and therefore display discrete contacts reminiscent of those of peri-centromeric contacts, Chromosight was able to discriminate between those two patterns, detecting specifically inter-centromeric interactions. The program was therefore able to correctly assess the subtle geometrical differences between these two patterns. Overall, this analysis shows the ability of Chromosight to quickly detect any type of user-defined pattern. We anticipate that many more patterns will be added to the catalogue of visual patterns linked to different molecular mechanisms of chromosome architecture.

## Discussion

In this work, we present Chromosight, a computer vision program to detect 3D structures in chromosome contact maps. We show that Chromosight outmatches other programs designed to detect chromosome loops, and that it can be used to extract other biologically relevant patterns generated through different chromosome capture derivatives.

Chromosight is versatile and we expect that additional pattern configurations will be added by the community, such as stripes, bow-shaped patterns, patterns associated to misassemblies or structural variations (e.g. inversions, translocations...) or any pattern of interest that the user can propose. The approach could therefore be used to investigate structural rearrangements in cancer cells, for instance, although the sensitivity of the program to detect rearrangements taking place in only a fraction of a population of cells remains to be tested. Similarly, the potential of the approach to develop new Hi–C based genome scaffolding algorithms could also be explored in the future^50,51^. The program has a great flexibility that allows to work with diverse biological data and address different questions, either using the de novo calling mode or the quantification mode. For instance, the possibility of varying the size of the loop kernel allows to optimise it for different conditions: larger kernels are more tolerant to noisy data (Fig 3c) as they dampen the fluctuations whereas smaller kernels allow to detect loops very close to the main diagonal (Supplementary Fig. 7).

A possible extension of the present approach is the addition of an iterative feedback step to the general flowchart of the current algorithm. Indeed, the output pileup after the first run of detection can be reused in another iteration of detection on the same data. This step could allow a finer adaptation to the data and to detect patterns a little further away from the initial kernel while keeping the basic characteristics.

With decreasing sequencing costs, new experimental protocols and optimised methods for amplifying specific genomic regions, we expect that the folding of the genomes of many species will be investigated in the near future using chromosome contact techniques. The algorithmic approach we present here provides a computational and statistical framework for the discovery of new principles governing chromosome architecture.

## Methods

### Simulation of Hi–C matrices

Simulated matrices were generated using a bootstrap strategy based on Hi–C data from chromosome 5 of mitotic *S. cerevisiae*^7^ at 2 kb resolution. Three main features were extracted from the yeast contact data (Supplementary Fig. 1): the probability of contact as a function of the genomic distance (*P*(*s*)), the positions of borders detected by HicSeg v1.1^52^ and positions of loops detected manually on chromosome 5. Positions from loops and borders were then aggregated into pileups of 17 × 17 pixels. We generated 2000 simulated matrices of 289 × 289 pixels. A first probability map of the same dimension is generated by making a diagonal gradient from *P*(*s*) representing the polymer behaviour. For each of the 2000 generated matrices, two additional probability maps are generated. The first by placing several occurrences of the border pileup on the diagonal, where the distance between borders follows a normal distribution fitted on the experimental coordinates. The second probability map is generated by adding the loop kernel 2–100 pixels away from the diagonal with the constraint that it must be aligned vertically and horizontally with border coordinates. For each generated matrix, the product of the *P*(*s*), borders and loops probability maps is then computed and used as a probability law to sample contact positions while keeping the same number of reads as the experimental map. This simulation method is implemented in the script chromo_simul.py, which can be found on the github repository: https://github.com/koszullab/chromosight_analyses_scripts.

### Benchmarking

To benchmark precision, sensitivity and F1 score, the simulated Hi–C data set with known loop coordinates were used. Each algorithm was run with a range of 60-180 parameter combinations (Supplementary Fig. 2) on 2000 simulated matrices and F1 score was calculated on the ensemble of results for each parameter combination separately (Supplementary Table 1). For each software, scores used in the final benchmark (Fig. 1) are those from the parameter combination that yielded the highest F1 score.

For the performance benchmark, HiCCUPS and HOMER were excluded. The former because it runs on GPU, and the latter because it uses genomic alignments as input and is much slower. The dataset used is a published high coverage Hi–C library^36^ from human lymphoblastoid cell lines (GM12878). To compare RAM usage across programs, this dataset was subsampled at 10%, 20%, 30%, 40% and 50% contacts and the maximum scanning distance was set to 2 Mbp. To compare CPU time, all programs were run on the full dataset, at different maximum scanning distances, with a minimum scanning distance of 0 and all other parameters left to default. All programs were run on a single thread, on a Intel(R) Core(TM) i7-8700K CPU at 3.70 GHz with 32 GB of available RAM.

Software versions used in the benchmark are Chromosight v0.9.0, hicexplorer v3.3.1, cooltools v0.2.0, homer 4.10 and hiccups 1.6.2. Input data, scripts and results of both benchmarks are available on Zenodo (10.5281/zenodo.3742095)

### Preprocessing of Hi–C matrices

Chromosight accepts input Hi–C data in cool format^53^. Prior to detection, Chromosight balances the whole-genome matrix using the ICE algorithm^31^ to account for Hi–C associated biases. For each intrachromosomal matrix, the observed/expected contact ratios are then computed by dividing each pixel by the mean of its diagonal. This erases the diagonal gradient due to the power-law relationship between genomic distance and contact probability, thus emphasising local variations in the signal (Fig. 1b). Intra-chromosomal contacts above a user-defined distance are discarded to constrain the analysis to relevant scales and improve performances.

### Calculation of Pearson coefficients

Correlation coefficients are computed by convolving the template over the contact map. Convolution algorithms are often used in computer vision where images are typically dense. Hi–C contact maps, on the other hand, can be very sparse. Chromosight’s convolution algorithm is therefore designed to be fast and memory efficient on sparse matrices. It can also exclude missing bins when computing correlation coefficients. Those bins appear as white lines on Hi–C matrices and can be caused by repeated sequences or low coverage regions.

The contact map can be considered an image IMG_CONT_ where the intensity of each pixel IMG_CONT_[*i*, *j*] represents the contact probability between loci *i* and *j* of the chromosome. In that context, each pattern of interest can be considered a template image IMG_TMP_ with *M*_TMP_ rows and *N*_TMP_ columns.

The correlation operation consists in sliding the template (IMG_TMP_) over the image (IMG_CONT_) and measuring, for each template position, the similarity between the template and its overlap in the image. We used the Pearson correlation coefficient as a the measure of similarity between the two images. The output of this matching procedure is an image of correlation coefficients IMG_CORR_ such that

1 $${{\rm{IMG}}}_{{\rm{CORR}}}[i,j]={\rm{Corr}}\,\left({{\rm{IMG}}}_{{\rm{CONT}}}\left[i-\frac{{M}_{{\rm{TMP}}}}{2}:i+\frac{{M}_{{\rm{TMP}}}}{2},j-\frac{{N}_{{\rm{TMP}}}}{2}:j+\frac{{N}_{{\rm{TMP}}}}{2}\right],\ {{\rm{IMG}}}_{{\rm{TMP}}}\right)$$

where the correlation operator *Corr*( ⋅ , ⋅ ) is defined as

2 $${\rm{Corr}}\,\left({{\rm{IMG}}}_{{{X}}},{{\rm{IMG}}}_{{{Y}}}\right)	= \frac{{\rm{cov}}\,({{\rm{IMG}}}_{{{X}}},{{\rm{IMG}}}_{{{Y}}})}{{\rm{std}}\,({{\rm{IMG}}}_{{{X}}})\cdot {\rm{std}}\,({{\rm{IMG}}}_{{{Y}}})}\\ 	=\frac{{\mathop {\sum} \limits _{(m,n)\in X\cap Y}}({{\rm{IMG}}}_{{{X}}}[m,n]-\overline{{{\rm{IMG}}}_{{{X}}}})\cdot ({{\rm{IMG}}}_{{{Y}}}[m,n]-\overline{{{\rm{IMG}}}_{{{Y}}}})}{\sqrt{{\mathop {\sum} \limits_{(m,n)\in X\cap Y}}{({{\rm{IMG}}}_{{{X}}}[m,n]-\overline{{{\rm{IMG}}}_{{{X}}}})}^{2}}\cdot \sqrt{{\mathop {\sum} \limits_{(m,n)\in X\cap Y}}{({{\rm{IMG}}}_{{{Y}}}[m,n]-\overline{{{\rm{IMG}}}_{{{Y}}}})}^{2}}}$$

where $\overline{{\rm{IMG}}}=\frac{1}{| X\cap Y| }\sum _{(m,n)\in X\cap Y}{\rm{IMG}}[m,n]$, *X* ∩ *Y* is the set of pixel coordinates that are valid in image IMG_*X*_ and in image IMG_*Y*_, and ∣*X* ∩ *Y*∣ is the number of valid pixels in IMG_*X*_ and IMG_*Y*_. A pixel in IMG_CONT_ is defined as valid when it is outside a region with missing bins.

### Separation of high-correlation foci

Selection is done by localising specific local maxima within IMG_CORR_. We proceeded as follows: first, we discard all points (*i*, *j*) where IMG_CORR_[*i*, *j*] < *τ*_CORR_. An adjacency graph *A*_*dxd*_ is then generated from the *d* remaining points. The value of *A*[*i*, *j*] is a boolean indicating the (four-way) adjacency status between the *i*th and *j*th nonzero pixels. The scipy implementation of the CCL algorithm for sparse graphs^54^ is then used on *A* to label the different contiguous foci of nonzero pixels. Foci with less than two pixels are discarded. For each focus, the pixel with the highest coefficient is determined as the pattern coordinate.

Patterns are then filtered out if they overlap too many empty pixels or are too close from another detected pattern. The remaining candidates in IMG_CORR_ are scanned by decreasing order of magnitude: every time a candidate is appended to the list of selected local maxima, all its neighbouring candidates are discarded. The proportion of empty pixels allowed and the minimum separation between two patterns are also user defined parameters.

### Biological analyses

Pairs of reads were aligned independently using Bowtie2 (v2.3.4.1) with --very-sensitive-local against the *S. cerevisiae* SC288 reference genome (GCF000146045.2). Uncuts, loops and religation events were filtered as described in ref. ^55^. Contact data were binned at 2 kb and normalised using the ICE balancing method^31^. Hi–C matrices were generated from fastq files using hicstuff v2.3.0^56^. Detection for biological analyses of yeast and human data was performed with default parameters using a 7 × 7 loop kernel available in Chromosight using --pattern loops_small unless mentioned otherwise. For enrichment analysis, cohesin peaks were defined using ChIP-seq data from^57^. Raw reads were aligned with bowtie2 and only mapped positions with Mapping Quality superior to 30 were kept and signals were also binned at 2 kb to synchronise with Hi–C data. Peaks of cohesins were considered with ChIP/input  >  1.5 and peaks closer than 10 kb to centromeres or rDNA were removed.

Annotation of highly expressed genes was done using RNA-seq data from^8^. Alignment was done as above. The distribution of the number of reads for each 2 kb bin was computed and the top 20% of the distribution were considered bins with high transcription. For border annotation, a set of plus or minus 1 bin on the detected positions is used. For human data, hg19 genome assembly was used with same strategy for alignment, construction and normalisation of contact data. ChIPseq peaks were retrieved from UCSC database (Supplementary Table 2). *B. subtilis* data were aligned with the PY79 genome version and the SMC signal was extracted using ChIP-chip data from^58^ and processed as described previously^10,59^. Peaks were annotated with the find_peaks function from scipy (v1.4.1), with parameters threshold = 0.1, width = 50. ChIA-PET data were processed as Hi–C data except that the contact maps were binned at a 500bp resolution. Epstein-Barr virus (EBV) genome, strain B95-8 (V01555.2) sequence was used to align the reads from EBV. For the detection in the different proximity ligation protocols, we retrieved publicly available data sets from the 4D Nucleome Data Portal^41^, and applied loops detection in the resulting contact maps of the mcool files at 10 kb resolution with the default settings by possibly changing one option that is indicated in (Fig. 4a).

### Reporting summary

Further information on research design is available in the Nature Research Reporting Summary linked to this article.

## Supplementary information

Supplementary Information

Peer Review

Reporting Summary

## Data Availability

All data associated with this study are publicly available and their reference numbers are listed in Supplementary Tables 2 and 3. Intermediate results, benchmark code and data are available on Zenodo (10.5281/zenodo.3742095).

## Code availability

Software and documentation available at https://github.com/koszullab/chromosight. All scripts required to reproduce figures and analyses are available at https://github.com/koszullab/chromosight_analyses_scripts.
