# Supplementary material for: Computer vision for pattern detection in chromosome contact maps
Source: Nat Commun. 2020 Nov 16;11:5795. doi: 10.1038/s41467-020-19562-7 (PMC7670471; doi:10.1038/s41467-020-19562-7)
Supplement: Supplementary file 1 — Supplementary Information [file 41467_2020_19562_MOESM1_ESM.pdf]

Supplementary information: Computer vision for pattern detection in chromosome contact maps

Cyril Matthey-Doret[1,2], Lyam Baudry[1,3,♮], Axel Breuer[4,♮], Rémi Montagne[1,3], Nadège Guiglielmoni[1,3], Vittore Scolari[1,3], Etienne Jean[1,3], Arnaud Campeas[4], Philippe Henri Chanut[4], Edgar Oriol[4], Adrien Meot[4], Laurent Politis[4], Antoine Vigouroux[5], Pierrick Moreau[1,3], Romain Koszul[1,3,*], Axel Cournac[1,3,*]

Supplementary Note 1

Fast 2D-convolution via SVD

Optionally, Chromosight's convolution algorithm can be accelerated further by approximating the template. This is done using truncated singular value decomposition (tSVD) to decompose the template into two sets of vectors whose product contain most of the information in the template, while reducing the number of operations needed in the convolution. This note explains the acceleration of 2D-convolution by using the SVD decomposition of the kernel. It was inspired from section 6.4.2 in *Computer and Robot Vision Vol. I* by Haralick and Shapiro (1992) [1].

The general case

Suppose that the contact map $\mathrm{IMG}_{\mathrm{CONT}}$ and the template $\mathrm{IMG}_{\mathrm{TMP}}$ have respectively size $(M_{\mathrm{CONT}}, N_{\mathrm{CONT}})$ and $(M_{\mathrm{TMP}}, N_{\mathrm{TMP}})$.

The convolution of $\mathrm{IMG}_{\mathrm{CONT}}$ by $\mathrm{IMG}_{\mathrm{TMP}}$, noted $\mathrm{IMG}_{\mathrm{CONT}} * \mathrm{IMG}_{\mathrm{TMP}}$, is an array such that

$$\left(\mathrm{IMG}_{\mathrm{CONT}} * \mathrm{IMG}_{\mathrm{TMP}}\right)[i,j] := \sum_{m=0}^{M_{\mathrm{TMP}}-1} \sum_{n=0}^{N_{\mathrm{TMP}}-1} \mathrm{IMG}_{\mathrm{CONT}}\left[i+m, j+n\right] \times \mathrm{IMG}_{\mathrm{TMP}}[m,n] \tag{1}$$

for $i = 1, \ldots, M_{\mathrm{CONT}} - M_{\mathrm{TMP}} + 1$ and $j = 1, \ldots, N_{\mathrm{CONT}} - N_{\mathrm{TMP}} + 1$. Otherwise stated $\mathrm{IMG}_{\mathrm{CONT}*\mathrm{TMP}} := \mathrm{IMG}_{\mathrm{CONT}} * \mathrm{IMG}_{\mathrm{TMP}}$ is an array of size $(M_{\mathrm{CONT}} - M_{\mathrm{TMP}} + 1, N_{\mathrm{CONT}} - N_{\mathrm{TMP}} + 1)$.

The computation of $\left(\mathrm{IMG}_{\mathrm{CONT}} * \mathrm{IMG}_{\mathrm{TMP}}\right)[i,j]$ requires $2\, M_{\mathrm{TMP}}\, N_{\mathrm{TMP}}$ operations, composed of $M_{\mathrm{TMP}}\, N_{\mathrm{TMP}}$ additions and $M_{\mathrm{TMP}}\, N_{\mathrm{TMP}}$ multiplications.

The separable case

Suppose that the template is *separable* i.e. there exists two vectors $\mathrm{U}_{\mathrm{TMP}}$ and $\mathrm{V}_{\mathrm{TMP}}$, with respective size (M_{TMP}) and (N_{TMP}), such that

$$\mathrm{IMG}_{\mathrm{TMP}}[m,n] = \mathrm{U}_{\mathrm{TMP}}[m]\, \mathrm{V}_{\mathrm{TMP}}[n]. \tag{2}$$

The operations in equation (1) can then be re-arranged more efficiently:

$$\begin{aligned}
\left(\mathrm{IMG}_{\mathrm{CONT}} * \mathrm{IMG}_{\mathrm{TMP}}\right)[i,j] &= \sum_{m=0}^{M_{\mathrm{TMP}}-1} \left(\sum_{n=0}^{N_{\mathrm{TMP}}-1} \mathrm{IMG}_{\mathrm{CONT}}\left[i+m, j+n\right] \times \mathrm{V}_{\mathrm{TMP}}[n] \right) \times \mathrm{U}_{\mathrm{TMP}}[m] && (3) \\
&= \sum_{m=0}^{M_{\mathrm{TMP}}-1} \mathrm{IMG}_{\mathrm{CONT}*\mathrm{V}_{\mathrm{TMP}}}[i+m, j] \times \mathrm{U}_{\mathrm{TMP}}[m] && (4)
\end{aligned}$$

where

$$\mathrm{IMG}_{\mathrm{CONT}*\mathrm{V}_{\mathrm{TMP}}}[i,j] := \sum_{n=0}^{N_{\mathrm{TMP}}-1} \mathrm{IMG}_{\mathrm{CONT}}\left[i+m, j+n\right] \times \mathrm{V}_{\mathrm{TMP}}[n] \tag{5}$$

The computation of an element $\mathrm{IMG}_{\mathrm{CONT}*\mathrm{TMP}}[i,j]$ costs N_{TMP} multiplications and N_{TMP} additions. According to equation (4), the computation of $\mathrm{IMG}_{\mathrm{CONT}*\mathrm{V}_{\mathrm{TMP}}}[i,j]$ requires $M_{\mathrm{TMP}} + N_{\mathrm{TMP}}$ multiplications and $M_{\mathrm{TMP}} + N_{\mathrm{TMP}}$ additions.

Consequently, the evaluation of $\mathrm{IMG}_{\mathrm{CONT}*\mathrm{TMP}}[i,j]$ costs $2\,(M_{\mathrm{TMP}} + N_{\mathrm{TMP}})$ operations in the separable case, which compares favorably to the $2\,M_{\mathrm{TMP}}\,N_{\mathrm{TMP}}$ operations required in the general case.

The SVD case

Next, suppose that the template has a representation as the sum of K separable kernels:

$$\mathrm{IMG}_{\mathrm{TMP}}[m,n] = \sum_{k=1}^{K} \mathrm{U}_{\mathrm{TMP}}[m,k]\,\mathrm{V}_{\mathrm{TMP}}[k,n]. \tag{6}$$

The number of operations involved in evaluating $\mathrm{IMG}_{\mathrm{CONT}*\mathrm{TMP}}[i,j]$ is $2\,(M_{\mathrm{TMP}} + N_{\mathrm{TMP}})$ for each kernel plus $K-1$ additions necessary to sum up the contribution of each kernel. In total, there are hence $2\,K\,(M_{\mathrm{TMP}} + N_{\mathrm{TMP}}) + K - 1$ operations.

The template $\mathrm{IMG}_{\mathrm{TMP}}$ is not necessarily equal to the superposition of K separable kernels, but it can always be approximated by such a superposition. The (truncated) SVD algorithm discussed below allows to construct such an approximation.

The Singular Value Decomposition (SVD) factorizes any rectangular matrix A of size (M,N) as

$$A = U\,D\,V \tag{7}$$

where U is a (M,M) orthogonal matrix, V is a (N,N) orthogonal matrix and D is a (M,N) matrix all of whose nonzero entries are on the diagonal and are positive.

Given any template $\mathrm{IMG}_{\mathrm{TMP}}$, it is possible to approximate it by retaining only the K largest singular values in the SVD of $A = \mathrm{IMG}_{\mathrm{TMP}}$, such that:

$$\mathrm{U}_{\mathrm{TMP}}[:,k] = \sqrt{D[k,k]}\,U[:,k] \tag{8}$$
$$\mathrm{V}_{\mathrm{TMP}}[k,:] = \sqrt{D[k,k]}\,V[k,:] \tag{9}$$
$$\tag{10}$$

Let's give a toy example of the operations spared by using a SVD approach. Suppose that $M_{\mathrm{TMP}} = N_{\mathrm{TMP}} = 17$, the standard convolution would require $2 \times 17 \times 17 = 578$ operations per point. In contrast, if we use a SVD convolution with $K = 1$, the number of operations reduces to 68, which represents only 12% of the brute force approach. Even with $K = 8$, we are below 50% of the brute force approach.

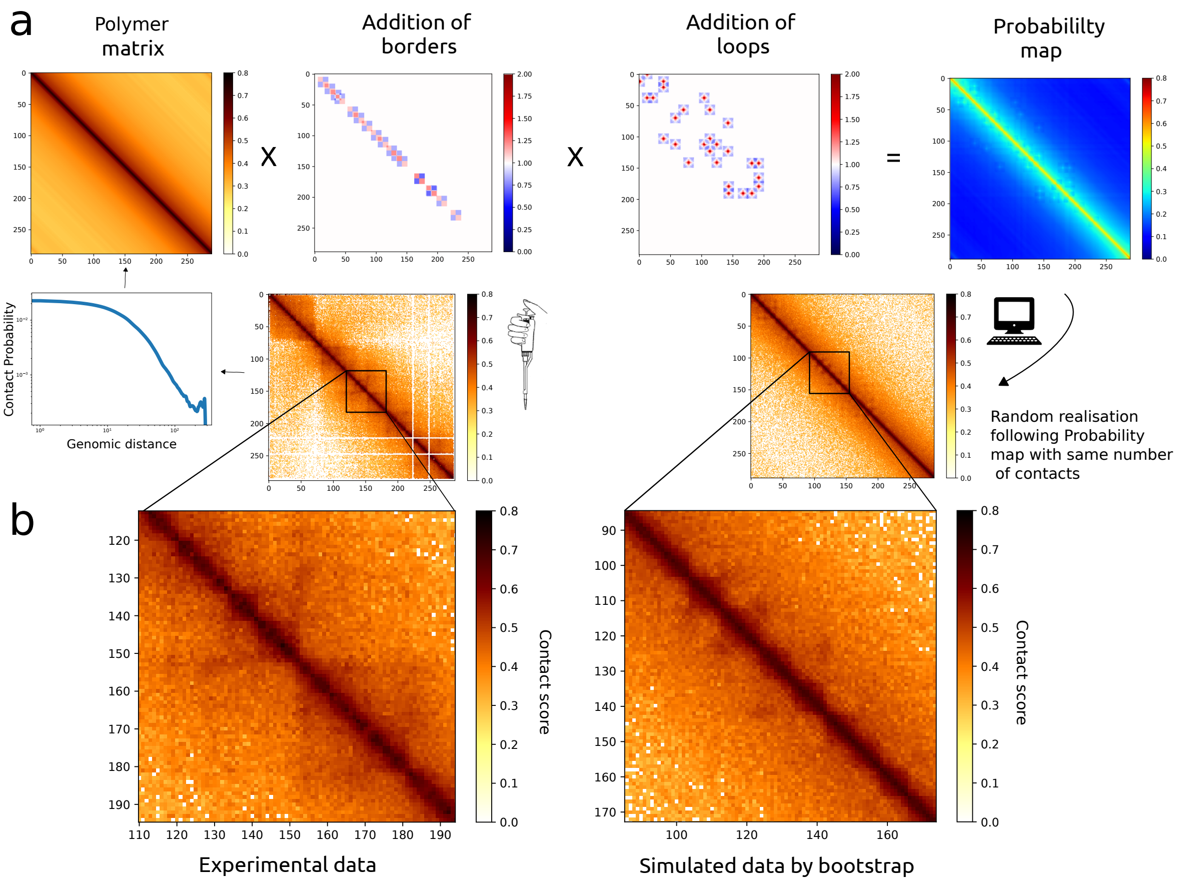

Supplementary Figure 1: Strategy for the generation of simulated contact data for benchmark tests of different loop calling algorithms a, The simulated data were generated with a bootstrap approach based on contact data generated for yeast *S. cerevisiae* in mitotic phase [2]. Three main features of the contact data were extracted: the probability of contact as a function of the genomic distance (P(s), Polymer matrix), presence of borders and presence of loops. The positions and intensity of border and loop patterns were defined thanks to pile-up signals from patterns detected by eye on the contact maps. Their positions were chosen according to a law of probabilities based on experimental data (see Methods). The product of the 3 feature matrices results in a probability matrix (**a**, right). This matrix is used as a probability law to sample contact positions while keeping the same number of reads as the experimental map. **b**, Zoom of contact maps for experimental and simulated data showing patterns of loops and borders. (Icons: [3], Perhelion / Wikimedia Commons, CC-BY-SA-3.0.)

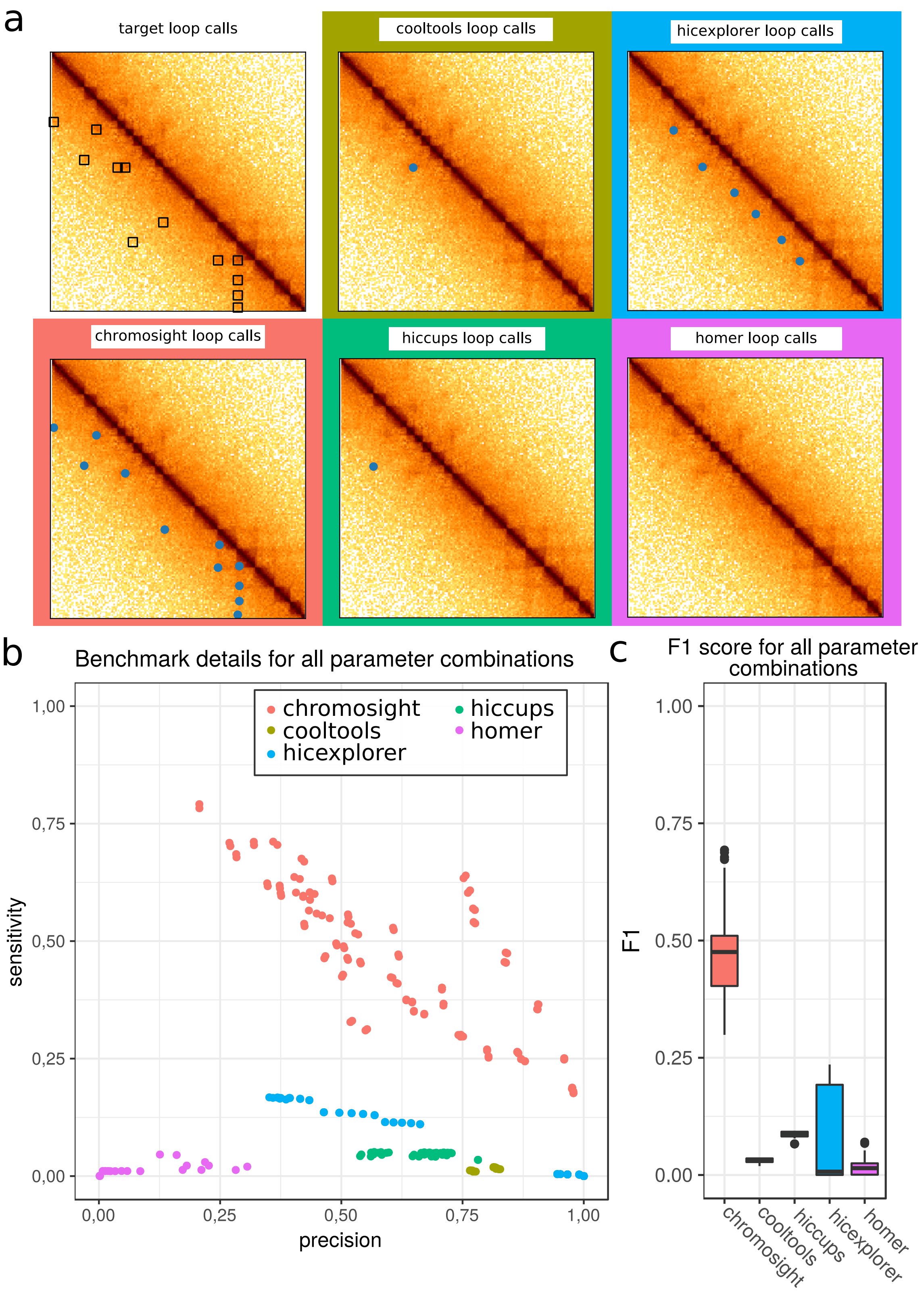

Supplementary Figure 2: Comparison of different loop callers on simulated data. **a**, Example region from a synthetic matrix with real loop calls (top left) and loops detected by all algorithms used in the benchmark using the combinations of parameters which yielded the highest F1 score. **b**, Precision and sensitivity for all algorithms on synthetic matrices, on the whole range of parameters tested. **c**, Distribution of F1 Scores for each algorithm for the range of parameters. Medians are shown as a black band inside boxplots. Hinges show the first and third quartiles and whiskers extend from the hinge to the furthest value within 1.5 times the inter-quartile distance (between first and third quartiles).

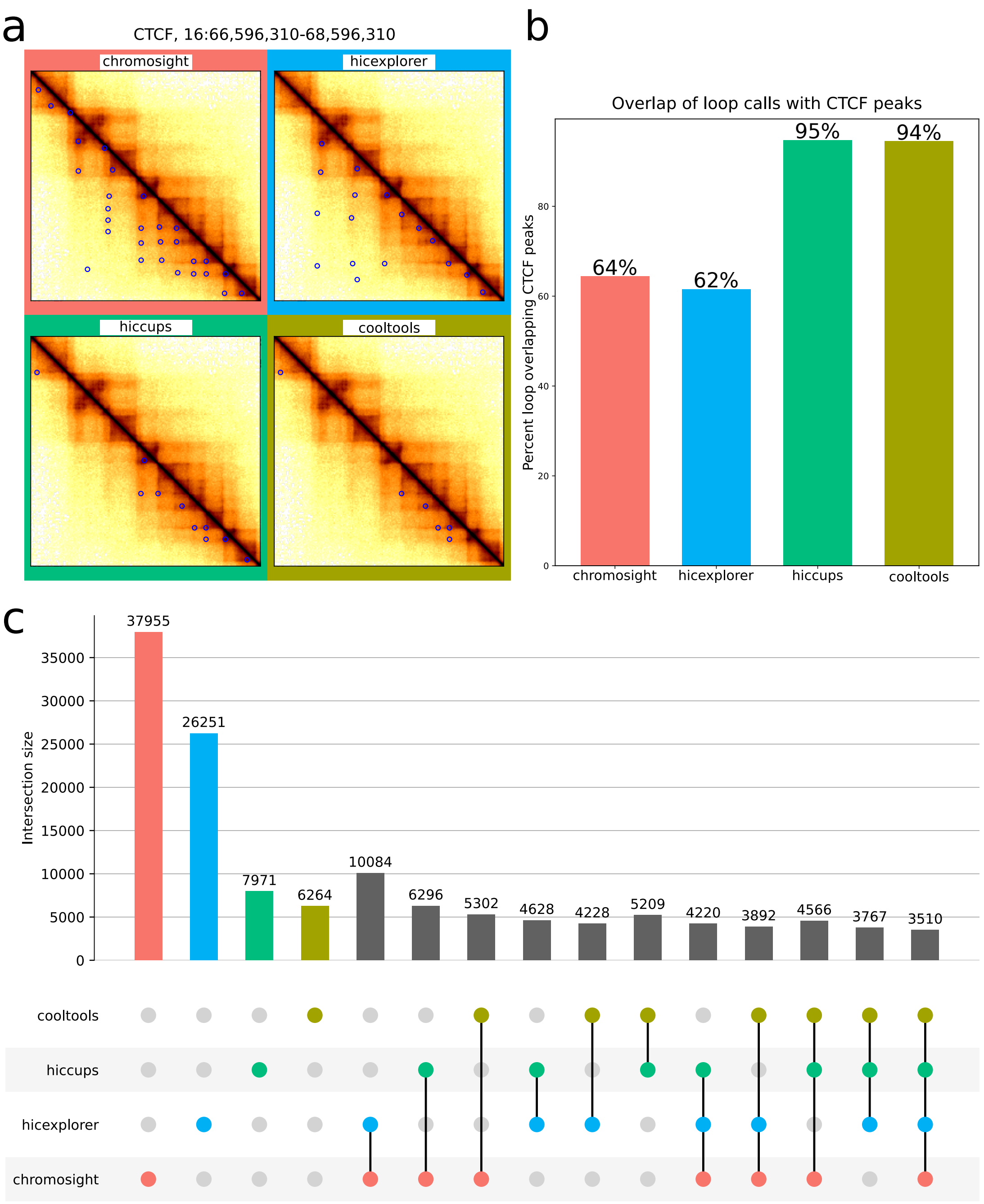

Supplementary Figure 3: Comparison of different loop callers on experimental data. **a**, Contact maps representating a region of +/- 1Mb around the CCCTC-binding factor (CTCF) gene of GM12878 (GSE63525, [4]), at 10kb resolution with coordinates of detected loops for different loop calling softwares, with default parameters. **b**, Proportion of loops with both anchors overlapping CTCF peaks [5]. An overlap is considered if loop anchors and CTCF peaks are within 10kb distance. **c**, Upsetplot showing the number of loops detected in GM12878 by each combination of softwares. Loops are considered identical if they are within 10kb of each other. For each combination of softwares, the intersection (∩) of detected coordinates is shown.

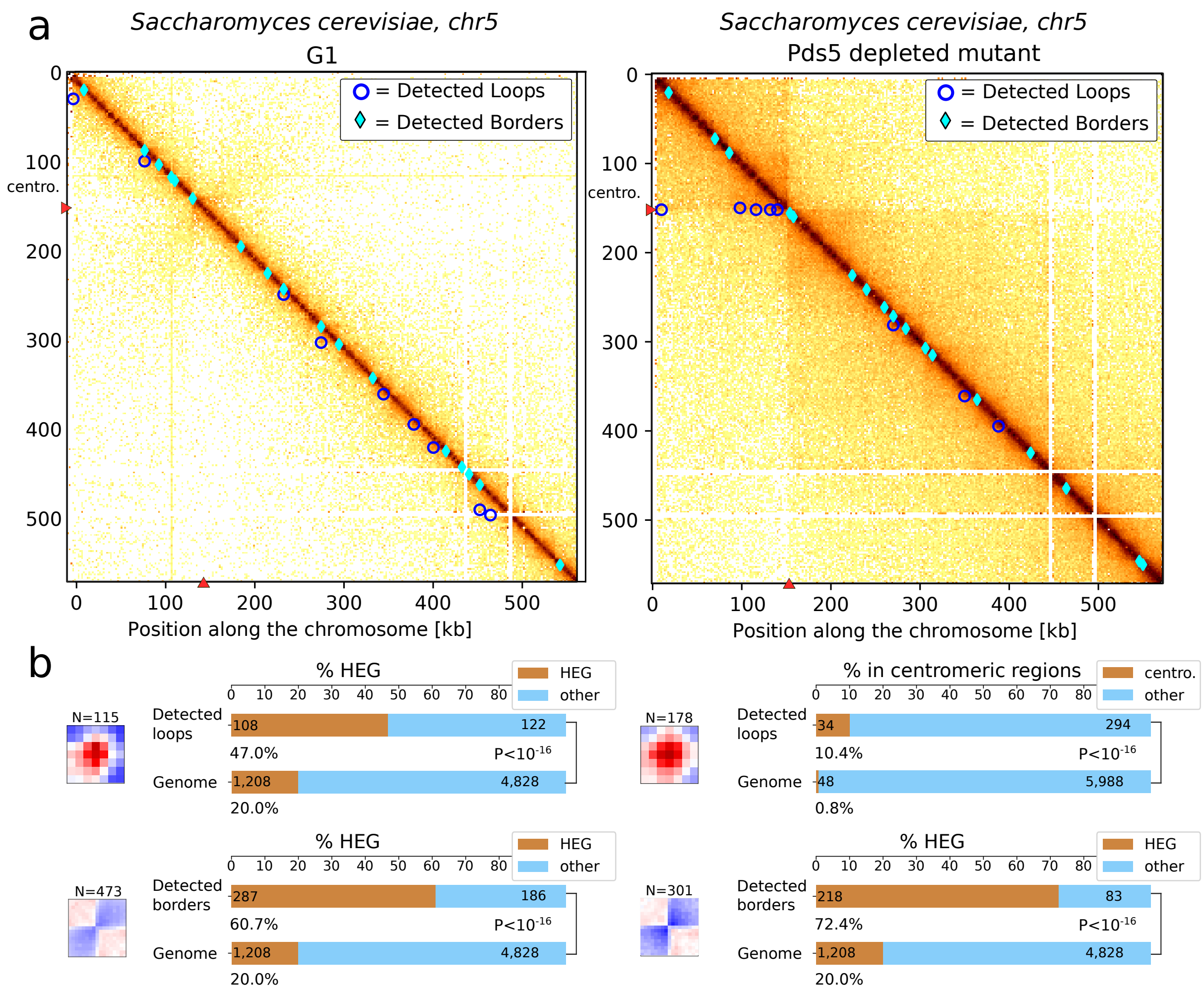

Supplementary Figure 4: Detection of loop and border patterns in yeast contact data a, Detection of loops and borders in Hi-C data of *S. cerevisiae* synchronised in G1 and for a mutant depleted in the protein Pds5 [2]. **b**, Bar plots showing enrichment in highly expressed genes (HEG) for detected loops in G1 and an enrichment in centromeric regions for the Pds5 mutant (Precocious Dissociation of Sisters gene). Bar plots showing enrichment in highly expressed genes for detected borders in G1 and Pds5 mutant. (Fisher test, two-sided)

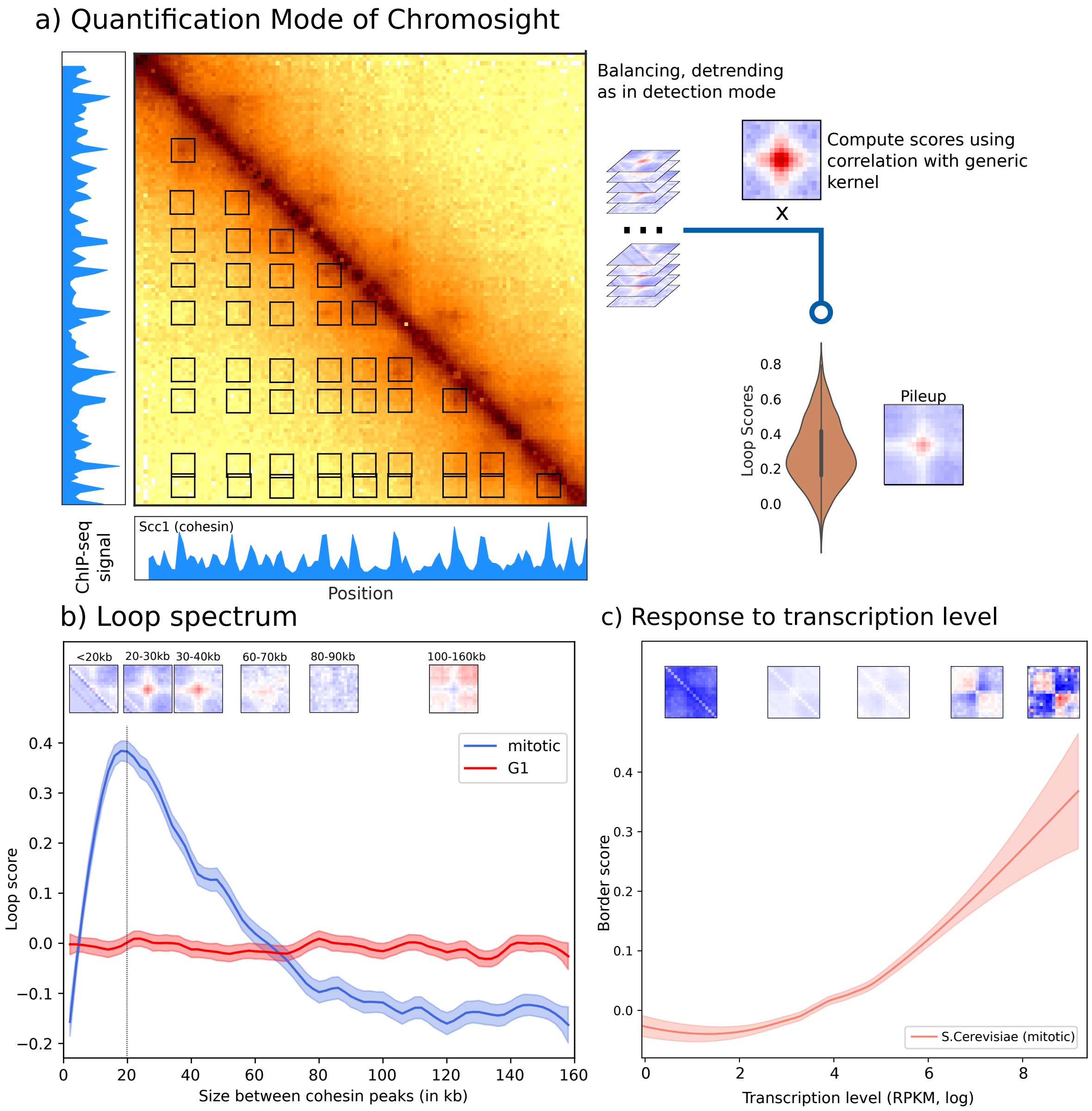

Supplementary Figure 5: Applications of quantification mode on yeast contact data a, Chromosight quantification mode workflow: sub-matrices from certain 2D genomic positions are extracted from balanced and detrended matrices (as in detection mode). Correlation with the kernel is then computed for each sub-matrix and the mean of all the sub-matrices is giving a pileup visualisation. Such 2D coordinates can be, for instance, pairs of protein enrichment peaks called from ChIP-seq data. **b**, Loop spectrum computed for the cohesin peaks network. The loop score is given as a function of distance between cohesin peaks for cells in mitotic state (data from [2]). Curves represent lowess-smoothed data with 95% confidence intervals. **c**, Plot showing the border score as a funtion of transcription levels in *S. cerevisiae*, (contact data and transcriptome data from [6]). The curve represents lowess-smoothed data with 95% confidence intervals.

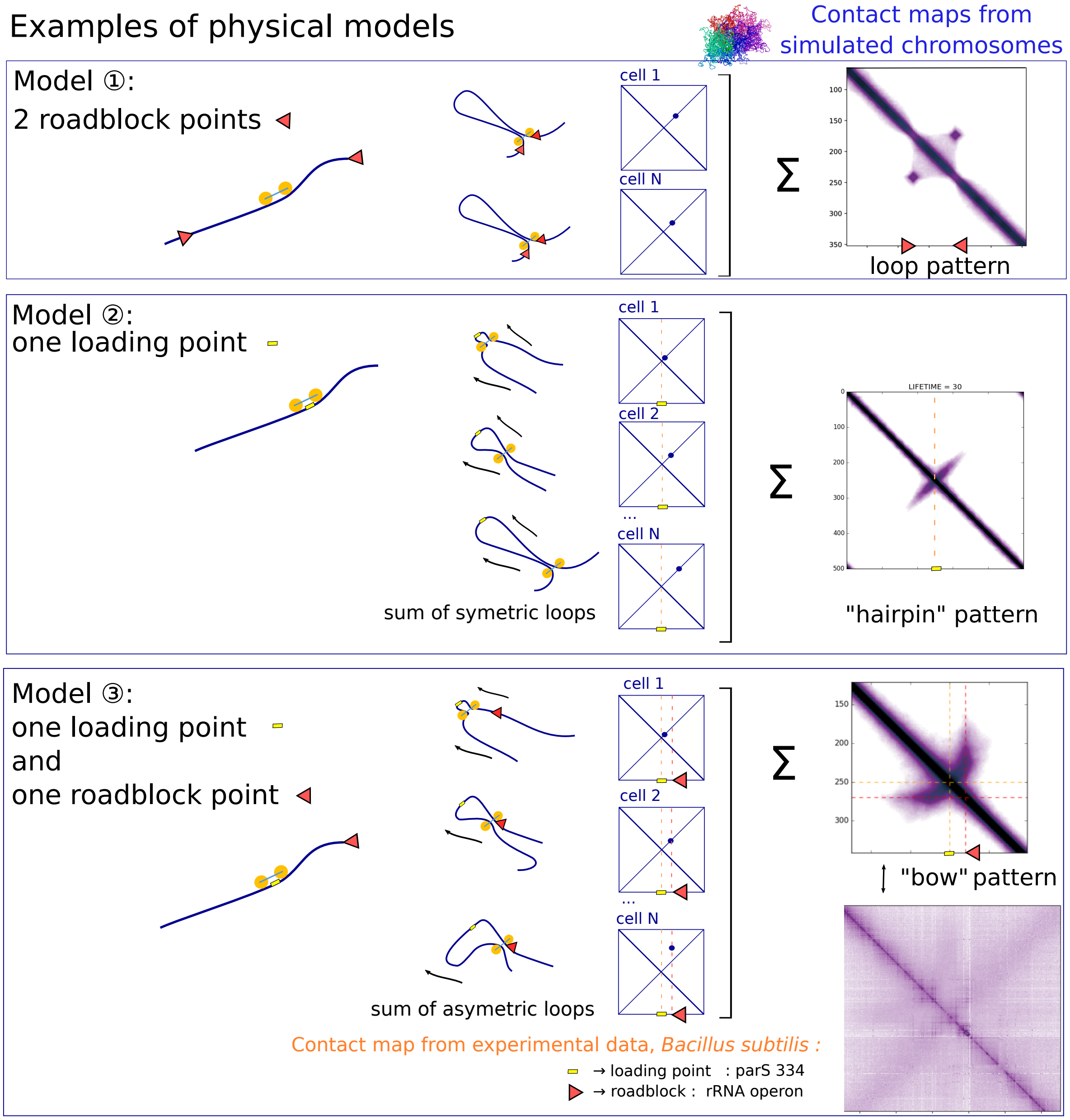

Supplementary Figure 6: Toy models that can link visual patterns and physical models. **Model 1**: loop extruding motors with two roadblock points leading to a loop pattern. **Model 2**: loop extruding motors with a specific loading point leading to a hairpin pattern. **Model 3**: loop extruding motors with a specific loading point and a single roadblock leading to a bow pattern. The bow pattern has been observed in contact data from *Bacillus subtilis* bacteria [7, 8]. By connecting the simulation and experimental contact data, the identified roadblock is a highly transcribed gene, (rDNA operon) and the loading site corresponds to the ParS 334 site. Molecular dynamics simulations were performed using OpenMM [9] and libraries with default parameters of [10].

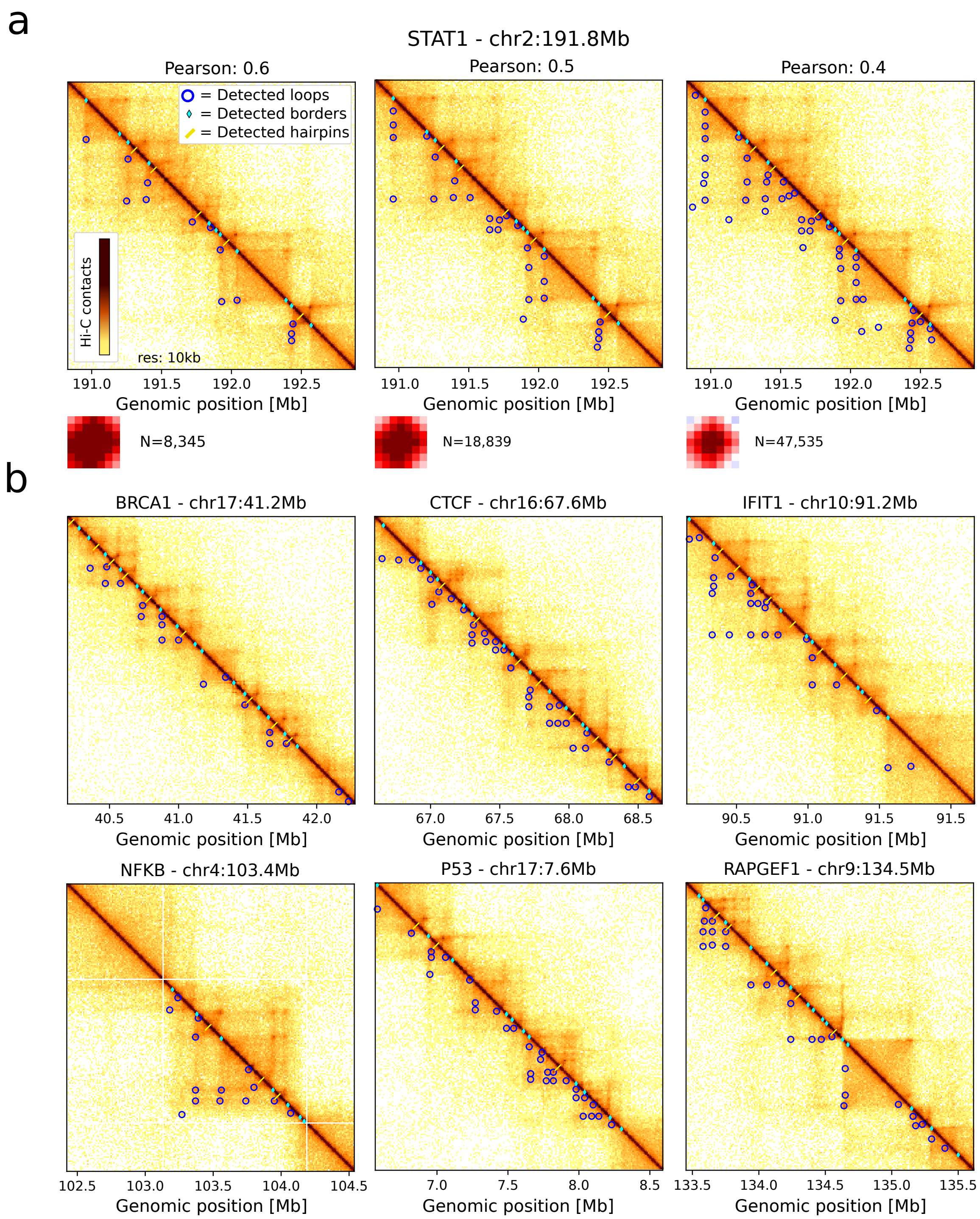

Supplementary Figure 7: Detection of loops, borders, hairpins in human Hi-C data. a, Effect of decreasing Chromosight's Pearson coefficient detection threshold on loop detection. Contact map in the vicinity of STAT1 gene in Hi-C data of lymphoblastoids [11] and total number of detected loops are shown for 3 Pearson threshold values: 0.6, 0.5, 0.4. Decreasing the Pearson threshold allows the detection of weak patterns. **b**, Zoom of contact maps 2 Mb around different genes of interest: BRCA1, CTCF, IFIT1, NFκB, P53, RAPGEF1 in Hi-C data of [11]. Detection done with Pearson coefficient parameter set to 0.5.

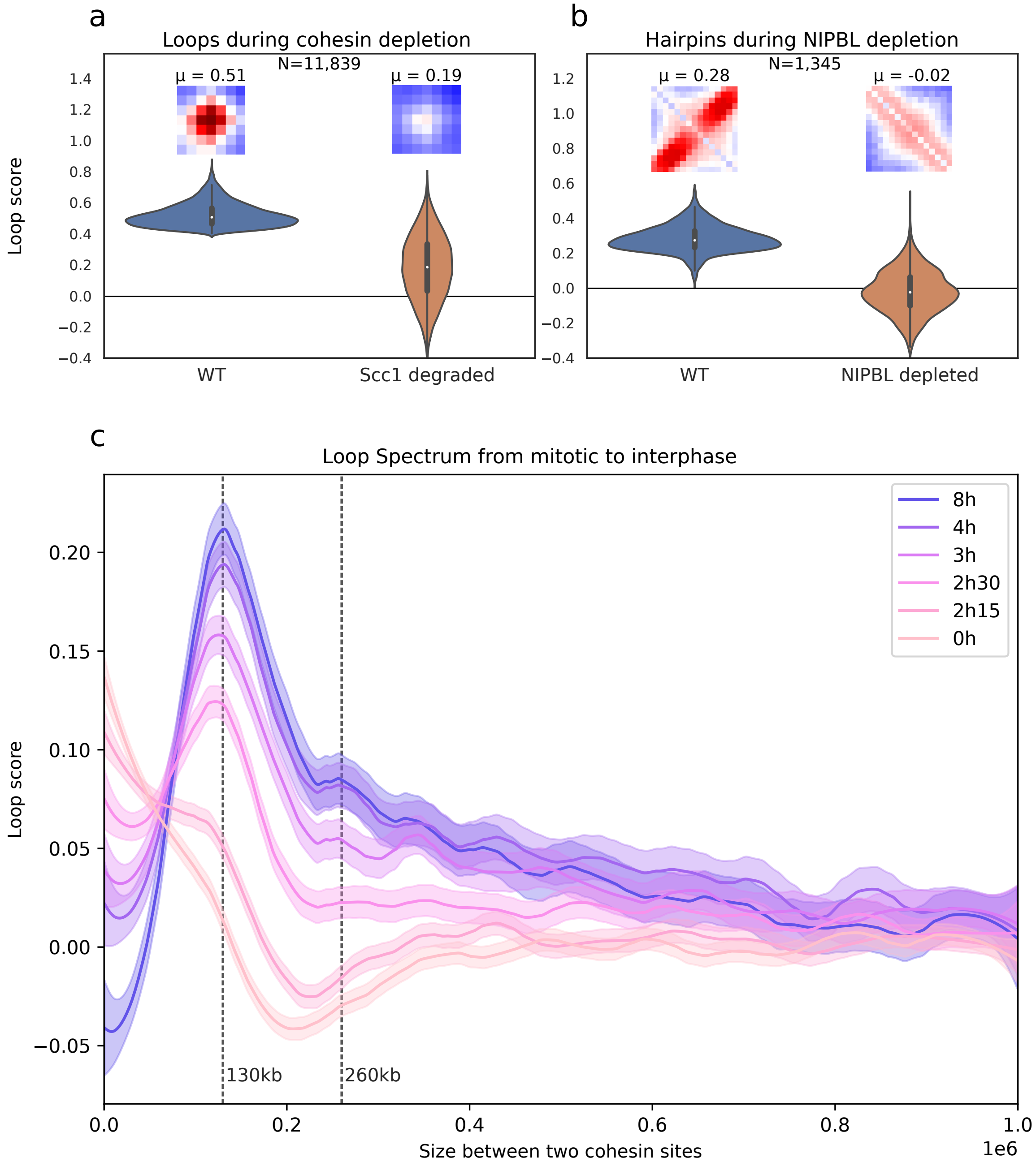

Supplementary Figure 8: Applications of quantification of loops and hairpins on human contact data. **a**, Comparison of loop score distributions in WT (*Homo sapiens*, HeLa cells) and in mutant cells depleted in Scc1 [12] for loops detected in WT condition. Associated pileup plots of windows centered on detected loops in WT condition. μ: median of loop scores. **b**, Comparison of hairpin score distributions in WT (*Mus musculus*, liver cells) and in mutant cells depleted in NIPBL [13] for hairpins detected in the WT condition. Associated pileup plots of windows centered on detected hairpins in WT condition. **c**, Loop spectrum showing correlation scores with the loop kernel for pairs of Rad21 ChIP-seq peaks separated by increasing distances, at different time points during release from mitosis into G1 (*Homo sapiens*, HeLa S3 cells) [14]. Curves represent lowess-smoothed data with 95% confidence intervals.

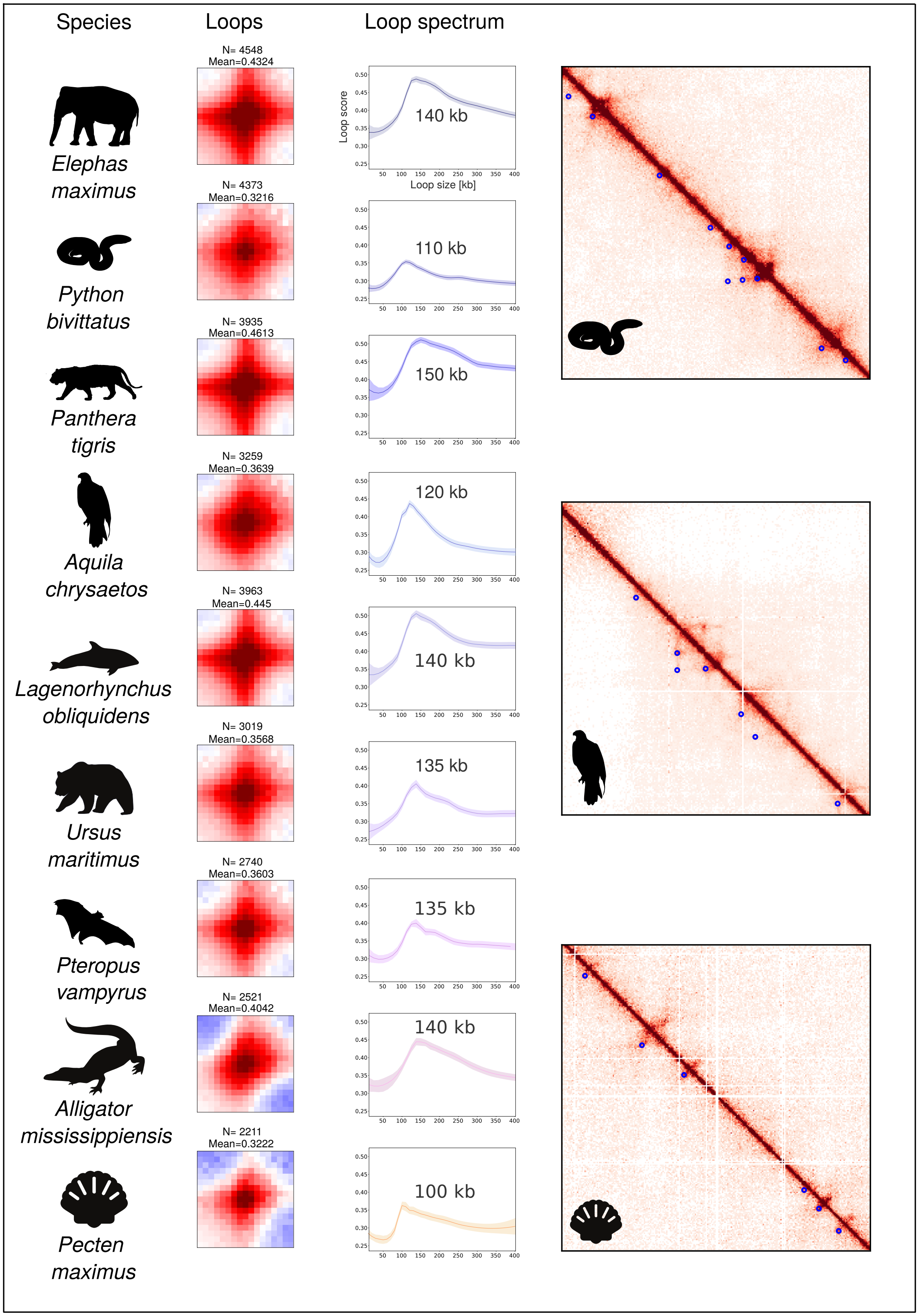

Supplementary Figure 9: Detection of loops, borders, hairpins in various animals from the DNA Zoo project [15]. From left to right, name of the species, associated pileup plots for the called loops and loop spectra computed on the positions of detected loops. The loop spectrum gives the size at which the detected loops have the highest scores. Curves represent lowess-smoothed data with 95% confidence intervals. Zooms on the right show examples of detected loops on Python bivittatus, Aquila chrysaetos and Pecten maximus, respectively. Detection has been performed on a standard laptop with a calculation time of less than 5 min for each pattern per organism. Credits for vectorized images: T. Michael Keesey (*Elephas maximus*), Steven Traver (*Panthera tigris*), Anthony Caravaggi (*Aquila chrysaetos*), Chris Huh (*Lagenorhynchus obliquidens*). Others are in the public domain and all are available on phylopic.org.

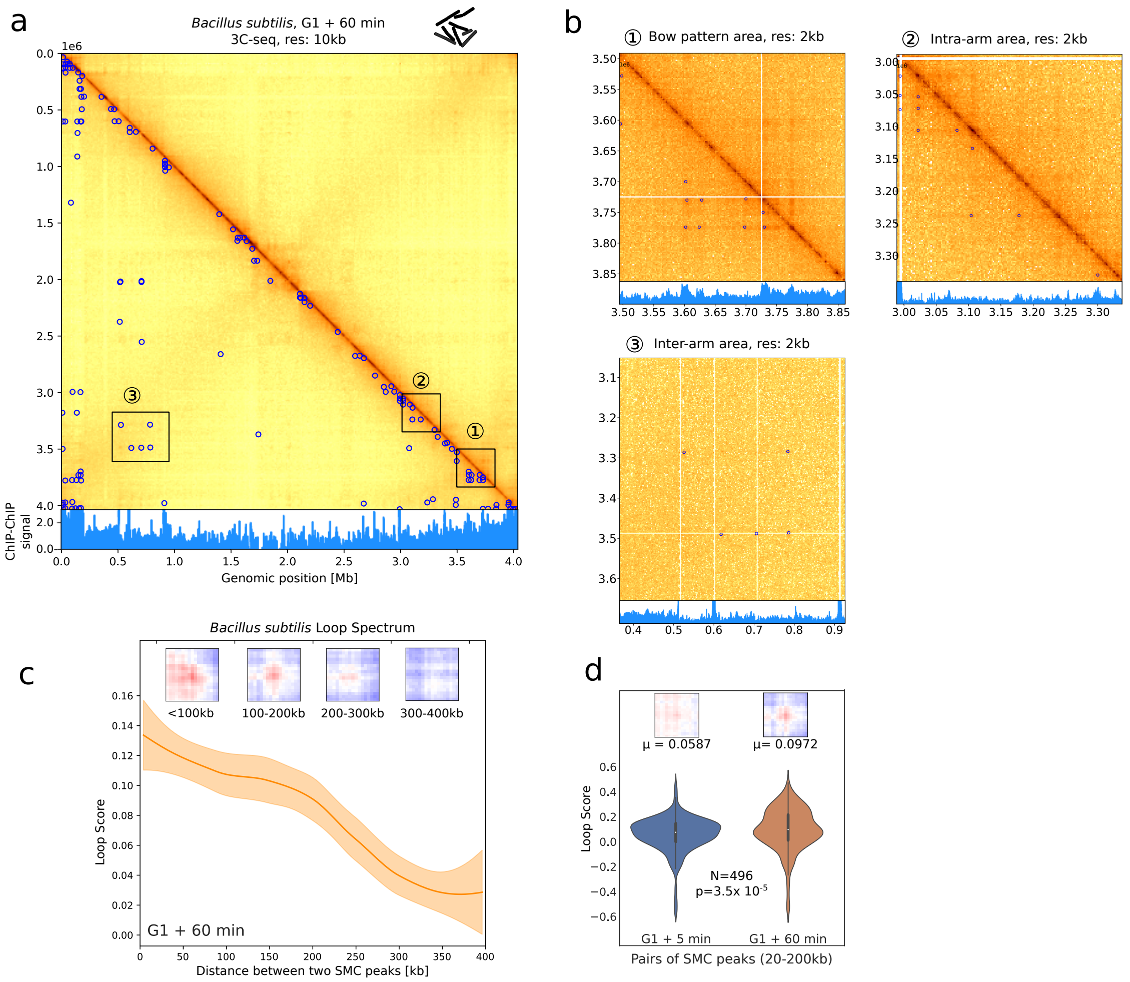

Supplementary Figure 10: Detection and quantification of loops in 3Cseq data of Bacillus subtilis. **a**, Detection of loops in 3Cseq data of *Bacillus subtilis* [16]. Genome contact map is shown at 10 kb resolution annotated with detected loops (carried on 2 kb data, 17x17 loop kernel). ChIP-chip signal of Structural Maintenance of Chromosomes proteins (SMC) is plotted under the map. Note that the origin of replication is at the end of the reference genome (bottom right of the contact map). **b**, Zooms of 3 genomic regions highlighted in panel a: in the bow pattern, in intra-arm region or in inter-arms area. **c**, Quantification of loop signal for pairs of SMC peaks for different sizes. Associated pileups of patterns for 4 size ranges are shown above. The curve represents lowess-smoothed data with 95% confidence intervals. **d**, Quantification of loop signals for pairs of SMC peaks between 20 and 200 kb in 2 conditions: G1 + 5 min and G1 + 60 min. Mean of loop scores and associated p-value (Paired Mann Withney U test, two-sided).

software	parameter	values	best F1
chromosight	--window-size	10,15,20	15
chromosight	--min-dist	0,40000	40000
chromosight	--pearson	0.30,0.35,0.40,0.45,0.50	0.30
chromosight	--min-separation	0,50000	0
hicexplorer	--windowSize	10,15,20	10
hicexplorer	--peakWidth	4,5,6,7,8	5
hicexplorer	--peakInteractionsThreshold	10,20,30	10
hicexplorer	--pValuePreselection	0.01,0.02,0.05,0.1	0.05
cooltools	--max-loci-separation	100000,200000,1000000,2000000	2000000
cooltools	--max-nans-tolerated	5,10,15,20	10
cooltools	--dots-clustering-radius	14000,19000,34000,39000	14000
hiccups	-p	1,2,4,6	1
hiccups	-i	6,10,14	14
hiccups	-f	0.05,0.1,0.2	0.1
homer	-poissonLoopGlobalBg	0.0001,0.001	0.001
homer	-poissonLoopLocalBg	0.01,0.05,0.1	0.05
homer	-window	2000,5000,10000	2000

Supplementary Table 1: Parameters used in the benchmark. Name and values of all parameters tested in the benchmark for each software. The best F1 column indicates which value yielded the best F1 score on the simulated dataset.

Organism	Experiment type	Figure	Ref	Identifier
S. cerevisiae	Hi-C, mitotic (nocodazole synchr.)	**Fig** 2	[6]	SRR7706226, SRR7706227
S. cerevisiae	Hi-C, G1 (alpha factor synchr.)	**Fig** 2	[2]	SRR8769554
S. pombe	Hi-C, Mitotic phase, 40 min	**Fig** 2	[17]	SRR5149256
S. cerevisiae	Hi-C, Pds5 depleted, mitotic (cdc20 synchr.)	**Sup Fig** 4	[2]	SRR8769553
H. sapiens	Hi-C, GM12878, asynchronous	**Fig** 3	[11]	SRR6675327
H. sapiens	Hi-C, HeLa cells, WT	**Sup Fig** 8	[12]	GSM2747745
H. sapiens	Hi-C, HeLa cells, depleted in Scc1	**Sup Fig** 8	[12]	GSM2747747
M. musculus	Hi-C, liver cells	**Sup Fig** 8	[13]	GSE93431
M. musculus	Hi-C, liver cells, depleted in NIPBL	**Sup Fig** 8	[13]	GSE93431
H. sapiens	Hi-C, HeLa cells during cell cycle (R2, T0, T2h15, T2h30, T3h, T4h, T8h)	**Sup Fig** 8	[14]	GSM3909703 GSM3909697 GSM3909696 GSM3909694 GSM3909691 GSM3909686
B. subtilis	3Cseq in G1 + 60 min	**Fig** 3	[7]	SRR2214080
B. subtilis	3Cseq in G1 + 5 min	**Sup Fig** 10	[7]	SRR2214069
Epstein Barr Virus	ChIA-PET of CTCF in GM12878 cells	**Fig** 3	[18]	SRR2312566
H. sapiens	In situ ChiA-PET, GM12878, asynchronous	**Fig** 4	[19]	4DNFIMH3J7RW
H. sapiens	DNA SPRITE, GM12878, asynchronous	**Fig** 4	[20]	4DNFIUOOYQC3
H. sapiens	HiChIP , GM12878, asynchronous	**Fig** 4	[21]	GSE80820_HiChIP _GM_cohesin.hic
H. sapiens	Micro-C , hESC, asynchronous	**Fig** 4	[22]	4DNFI9FVHJZQ
C. albicans	Hi-C, asynchronous	**Fig** 5	[23]	SRR3381672
E. maximus	Hi-C, asynchronous	**Sup Fig** 9	[15]	Elephas_maximus rawchrom.hic
P. bivittatus	Hi-C, asynchronous	**Sup Fig** 9	[24]	Python_bivittatus rawchrom.hic
P. tigris	Hi-C, asynchronous	**Sup Fig** 9	[25]	Panthera_tigris rawchrom.hic
A. chrysaetos	Hi-C, asynchronous	**Sup Fig** 9	[26]	Aquila_chrysaetos rawchrom.hic
L. obliquidens	Hi-C, asynchronous	**Sup Fig** 9	[15]	Lagenorhynchus _obliquidens rawchrom.hic
U. maritimus	Hi-C, asynchronous	**Sup Fig** 9	[27]	Ursus_maritimus rawchrom.hic
P. vampyrus	Hi-C, asynchronous	**Sup Fig** 9	[28]	Pectorus_vampyrus rawchrom.hic
A. mississippiensis	Hi-C, asynchronous	**Sup Fig** 9	[29][30]	Alligator_mississi -ppiensis rawchrom.hic
P. maximus	Hi-C, asynchronous	**Sup Fig** 9	[31]	Pecten_maximus rawchrom.hic

Supplementary Table 2: Different contact datasets analysed in the present study. The last column indicates either the identifier for the raw reads available on the Short Read Archive server (SRA) (https://www.ncbi.nlm.nih.gov/sra), the identifier of the .cool files accessible on the Gene Expression Omnibus server (GEO) https://www.ncbi.nlm.nih.gov/geo or the name of hic files from DNA zoo project available on https://www.dnazoo.org/assemblies [15] from which the analysis were made. mcool files coming from 4DN portal were downloaded from the server https://data.4dnucleome.org [32].

Organism	Experiment type	Figure	Ref	Identifier
S. cerevisiae	RNA-seq, mitotic (nocodazole synchr.)	**Fig** 2	[6]	SRR7692240
S. cerevisiae	ChIP-seq, Scc1PK9 IP G1 releasing 60min	**Fig** 2	[33]	SRR2065097, SRR2065092
H. sapiens	ChIP-seq CTCF	**Fig** 3	[5]	wgEncodeAwgTfbsBroad Gm12878CtcfUniPk.narrowPeak
H. sapiens	ChIP-seq RAD21	**Fig** 3	[5]	wgEncodeAwgTfbsHaib Gm12878Rad21V0416101UniPk
H. sapiens	ChIP-seq NIPBL	**Fig** 3	[5]	GSM2443453_GM12878_NIPBL Rep1_ 2WCE_Narrow_Peaks_peaks.narrowPeak
B. subtilis	ChIP-chip of SMC	**Fig** 3	[34]	GSE14693
Epstein Barr Virus	ChIP-seq CTCF	**Fig** 3	[35]	SRR036682
Epstein Barr Virus	ChIP-seq RAD21	**Fig** 3	[18]	SRR2312570

Supplementary Table 3: Other genomic datasets used in the present study. The last column indicates either the identifier for the raw reads available on the Short Read Archive server (SRA) (https://www.ncbi.nlm.nih.gov/sra), the identifier of the ChIP-chip files accessible on the Gene Expression Omnibus server (GEO) https://www.ncbi.nlm.nih.gov/geo or the identifier of ChIP-seq peak files available on http://genome.ucsc.edu.

References

1. Haralick, R. M. & Shapiro, L. G. *Computer and Robot Vision* 1st. ISBN: 0201569434 (Addison-Wesley Longman Publishing Co., Inc., USA, 1992).

2. Dauban, L. *et al.* Regulation of Cohesin-Mediated Chromosome Folding by Eco1 and Other Partners. *Molecular Cell* **77,** 1279–1293.e4. https://doi.org/10.1016/j.molcel.2020.01.019 (Mar. 2020).

3. OpenWetWare. *BISC209/S13: Use and Care of Micropipets — OpenWetWare,* [Online; accessed 5-October-2020]. 2013. https://openwetware.org/mediawiki/index.php?title=BISC209/S13:_Use_and_Care_of_Micropipets&oldid=666811.

4. Rao, S. S. P. *et al.* A 3D map of the human genome at kilobase resolution reveals principles of chromatin looping. eng. *Cell* **159,** 1665–1680. ISSN: 1097-4172 (Dec. 2014).

5. Karolchik, D. The UCSC Table Browser data retrieval tool. *Nucleic Acids Research* **32,** 493D–496. https://doi.org/10.1093/nar/gkh103 (Jan. 2004).

6. Garcia-Luis, J. *et al.* FACT mediates cohesin function on chromatin. *Nat. Struct. Mol. Biol.* **26,** 970–979 (Oct. 2019).

7. Marbouty, M. *et al.* Condensin-and replication-mediated bacterial chromosome folding and origin condensation revealed by Hi-C and super-resolution imaging. *Molecular cell* **59,** 588–602 (2015).

8. Banigan, E. J., van den Berg, A. A., Brandão, H. B., Marko, J. F. & Mirny, L. A. Chromosome organization by one-sided and two-sided loop extrusion. *eLife* **9.** ISSN: 2050-084X. http://dx.doi.org/10.7554/eLife.53558 (Apr. 2020).

9. Eastman, P. *et al.* OpenMM 7: Rapid development of high performance algorithms for molecular dynamics. *PLOS Comp. Biol.13(7): e1005659.* (2017).

10. Goloborodko, A., Imakaev, M. V., Marko, J. F. & Mirny, L. Compaction and segregation of sister chromatids via active loop extrusion. *Elife* **5,** e14864 (2016).

11. Ghurye, J. *et al.* Integrating Hi-C links with assembly graphs for chromosome-scale assembly. *PLoS Comput. Biol.* **15,** e1007273 (Aug. 2019).

12. Wutz, G. *et al.* Topologically associating domains and chromatin loops depend on cohesin and are regulated by CTCF, WAPL, and PDS5 proteins. *EMBO J.* **36,** 3573–3599 (Dec. 2017).

13. Schwarzer, W. *et al.* Two independent modes of chromatin organization revealed by cohesin removal. *Nature* **551,** 51–56 (Nov. 2017).

14. Abramo, K. *et al.* A chromosome folding intermediate at the condensin-to-cohesin transition during telophase. *Nat. Cell Biol.* **21,** 1393–1402 (Nov. 2019).

15. Dudchenko, O. *et al.* De novo assembly of the Aedes aegypti genome using Hi-C yields chromosome-length scaffolds. *Science* **356,** 92–95. https://doi.org/10.1126/science.aal3327 (Mar. 2017).

16. Marbouty, M. *et al.* Metagenomic chromosome conformation capture (meta3C) unveils the diversity of chromosome organization in microorganisms. eng. *Elife* **3,** e03318. http://dx.doi.org/10.7554/eLife.03318 (2014).

17. Tanizawa, H., Kim, K.-D., Iwasaki, O. & Noma, K.-I. Architectural alterations of the fission yeast genome during the cell cycle. *Nat. Struct. Mol. Biol.* **24,** 965–976 (Nov. 2017).

18. Tang, Z. *et al.* CTCF-Mediated Human 3D Genome Architecture Reveals Chromatin Topology for Transcription. *Cell* **163,** 1611–27 (Dec. 2015).

19. Li, X. *et al.* Long-read ChIA-PET for base-pair-resolution mapping of haplotype-specific chromatin interactions. *Nature Protocols* **12,** 899–915. ISSN: 1750-2799. http://dx.doi.org/10.1038/nprot.2017.012 (Mar. 2017).

20. Quinodoz, S. A. *et al.* Higher-Order Inter-chromosomal Hubs Shape 3D Genome Organization in the Nucleus. *Cell* **174,** 744–757.e24. ISSN: 0092-8674. http://dx.doi.org/10.1016/j.cell.2018.05.024 (July 2018).

21. Mumbach, M. R. *et al.* HiChIP: efficient and sensitive analysis of protein-directed genome architecture. *Nature Methods* **13,** 919–922. ISSN: 1548-7105. http://dx.doi.org/10.1038/nmeth.3999 (Sept. 2016).

22. Krietenstein, N. *et al.* Ultrastructural Details of Mammalian Chromosome Architecture. *Molecular Cell* **78,** 554–565.e7. ISSN: 1097-2765. http://dx.doi.org/10.1016/j.molcel.2020.03.003 (May 2020).

23. Burrack, L. S. *et al.* Neocentromeres Provide Chromosome Segregation Accuracy and Centromere Clustering to Multiple Loci along a Candida albicans Chromosome. *PLOS Genetics* **12** (ed Mellone, B. G.) e1006317. ISSN: 1553-7404. http://dx.doi.org/10.1371/journal.pgen.1006317 (Sept. 2016).

24. Castoe, T. A. *et al.* The Burmese python genome reveals the molecular basis for extreme adaptation in snakes. *Proceedings of the National Academy of Sciences* **110,** 20645–20650. https://doi.org/10.1073/pnas.1314475110 (Dec. 2013).

25. Cho, Y. S. *et al.* The tiger genome and comparative analysis with lion and snow leopard genomes. *Nature Communications* **4.** https://doi.org/10.1038/ncomms3433 (Sept. 2013).

26. Bussche, R. A. V. D., Judkins, M. E., Montague, M. J. & Warren, W. C. A Resource of Genome-Wide Single Nucleotide Polymorphisms (Snps) for the Conservation and Management of Golden Eagles. *Journal of Raptor Research* **51,** 368–377. https://doi.org/10.3356/jrr-16-47.1 (Sept. 2017).

27. Liu, S. *et al.* Population Genomics Reveal Recent Speciation and Rapid Evolutionary Adaptation in Polar Bears. *Cell* **157,** 785–794. https://doi.org/10.1016/j.cell.2014.03.054 (May 2014).

28. Lindblad-Toh, K. *et al.* A high-resolution map of human evolutionary constraint using 29 mammals. *Nature* **478,** 476–482. https://doi.org/10.1038/nature10530 (Oct. 2011).

29. John, J. A. S. *et al.* Sequencing three crocodilian genomes to illuminate the evolution of archosaurs and amniotes. *Genome Biology* **13.** https://doi.org/10.1186/gb-2012-13-1-415 (Jan. 2012).

30. Rice, E. S. *et al.* Improved genome assembly of American alligator genome reveals conserved architecture of estrogen signaling. *Genome Research* **27,** 686–696. https://doi.org/10.1101/gr.213595.116 (Jan. 2017).

31. Kenny, N. J. *et al.* The Gene-Rich Genome of the Scallop Pecten maximus. https://doi.org/10.1101/2020.01.08.887828 (Jan. 2020).

32. Dekker, J. *et al.* The 4D nucleome project. *Nature* **549,** 219–226. https://doi.org/10.1038/nature23884 (Sept. 2017).

33. Hu, B. *et al.* Biological chromodynamics: a general method for measuring protein occupancy across the genome by calibrating ChIP-seq. *Nucleic Acids Research,* gkv670. https://doi.org/10.1093/nar/gkv670 (June 2015).

34. Gruber, S. & Errington, J. Recruitment of condensin to replication origin regions by ParB/SpoOJ promotes chromosome segregation in B. subtilis. eng. *Cell* **137,** 685–696. http://dx.doi.org/10.1016/j.cell.2009.02.035 (May 2009).

35. McDaniell, R. *et al.* Heritable Individual-Specific and Allele-Specific Chromatin Signatures in Humans. *Science* **328,** 235–239. https://doi.org/10.1126/science.1184655 (Mar. 2010).
