# Supplementary material for: Computer vision for pattern detection in chromosome contact maps
Source: Nat Commun. 2020 Nov 16;11:5795. doi: 10.1038/s41467-020-19562-7 (PMC7670471; doi:10.1038/s41467-020-19562-7)
Supplement: Supplementary file 2 — Peer Review [file 41467_2020_19562_MOESM2_ESM.pdf]

Reviewer #1 (Remarks to the Author):

The authors present an application of computer vision methods to detect specific contact patterns in HiC contact maps. The theme is of high relevance as more and more HiC datasets become available across different species and cell states. Undeniably, so far the study of these contact maps has been quite limited to the features that were initially identified such as TADs and compartments and despite the recognized importance of chromatin loops, it remains difficult to identify them against noise.
The method is particularly directed at the detection of loops but it can be also used to identify other patterns in the contact maps, which could be just as functionally relevant. Chromosight, their software, appears to be much faster and more accurate (if we consider sensitivity, which I believe is very important) compared to alternative existing methods.

It is mostly based on performing a convolution of a specific template with the contact map represented as an image, and thus is able to quantify the number of sites where the template is matched and the correlation between the template and the specific region of the contact map can be estimated.

The authors showcase their method on synthetic HiC data mimicking budding yeast. They then proceed to test enrichment of loops detected in budding yeast data in cohesin binding peaks but also show that some of the loops they call are different in mutants are lacking cohesin or having altered loop extrusion.

They then apply the method to human lymphoblastoid cell line GM12878 recovering loops, borders and hairpins structures that are consistent with enrichments of the regions in cohesin and CTCF, as expected. Investigating HiC contact maps from many different animals they identify a conserved loop preferred length of 100kb and they also detect loops from 3C seq of Bacilus Subtilis. Most interestingly, they also detect loops on ChIA-PET data from a virus, the existence of which had been proposed based on other types of observations, which also coincide with occupancy of cohesin and CRCT.

The detection of patterns with their tools is completely user-defined, as they provide a point and click mode of their software to identify templates of interest directly from the data. This can also be used to define a template in one species and recover matching features in another, as shown with pericentromeric inter-chromosomal contacts in 2 yeast species.

The documentation is well written, complete and easy to follow, with all data provided to the user. I installed the software and had no issues. I'm looking forward to using it!

Major Comments:
Generally, I found the paper extremely clear and well-written with clear and informative figures.
I believe there are two main points that could be improved (note that I am not an expert in yeast, bacterial or viral genomes):
Since the authors focus on the GM12878 cell line, whose chromatin contacts have been characterised by a variety of techniques, it would be particularly interesting to see if their loop detection (or indeed detection of other patterns such as hairpins) would be similar starting from the different datasets. Since the method is able to use virtually any chromosome capture technology as input and it is so efficient, I would have liked to see the results using HiC compared to at least some of the other available datasets for these cells (ChIA-PET, promoter Capture HiC, HiChIP, SPRITE).
More specifically I would be curious to know if the scale of these loops that they identify to peak at 100kb is confirmed across techniques, and if there are other (longer-distance) peaks in this distribution, which are hard to identify in human with HiC.

I think a little bit more background information should be given considering the wide readership of the

journal. Especially a few more sentences about the differences of chromosome structures across species would be welcome. The detection of hairpins and bow shape patterns is very interesting but I think the authors should give a few more details about the characteristics of these loops. I found the figures in supplementary helpful but I think a lengthier discussion of the concept could clarify these structures better and highlighted their importance. Also cohesin injection should be defined and discussed in the context of recent literature on loop extrusion. This could probably fit in the introduction, so the reader has the necessary background to understand the result, or in the discussion.
In line 108, some more words should be spent on the possibility that the loops identified corresponds to interacting replichores.

Maybe it would be good to run some comparison with other methods on a single chromosome of human data even though they are computationally intensive?

Minor issues:
Typos:
Line 80 analysis confirms
Line 81 specific moments
Line 137 which contains

Vera Pancaldi

Reviewer #2 (Remarks to the Author):

This manuscript introduces the authors' original computation program Chromosight for detecting chromatin structures with computer vision from omics data marking chromatin contacts, such as Hi-C data. While I am convinced by the value and power of the program, I find the manuscript attractive to only a specific range of readers and suitable to a specialized or methodological journal.

Please supply concrete size information in this part in the Introduction -'bacteria where the sizes of the structures are tiny compared to mammalian genomes'. How tiny are they? Overall, if the authors intend to publish this manuscript in a non-specialized journal, it is strongly recommended to include more biological and concrete information (e.g., SMC protein) in the Introduction as basic introductory items to later support their findings.

It is abrupt that in the middle of Introduction, Suppl Fig. 3a is cited without any indication that they performed an analysis to produce these data. As a solution, I would suggest not to cite any item in Supplementary Material in the Introduction.

The Abstract is not structured to support their conclusion. Especially, these sentences lack the method of comparison and validation. - 'Chromosight has greater sensitivity than existing methods, while being faster and applicable to any type of genomes, including bacteria, viruses, yeasts and mammals. Our results confirm the universal role of Structural Maintenance of Chromosomes proteins (SMC) in the formation of loops at the chromosome level.' Also, the content of the second sentence above is not worth including in the Abstract because it is not their original finding. If insisting on including it, the authors should qualify themselves to include this information just as a method for validating the superiority of their program but not more than that.

Code Availability section provides the link to the GitHub page for Chromosight, but does not include other items to reproduce the benchmarking, etc. presented in the manuscript.

Labeling of the horizontal axis for Figure 1b, c, and d is very confusing.

Some parts of the texts in the Figures are not consistent in terms of italic/non-italic and capital/lowercases choice. The abbreviation of the genus names is also inconsistent in some parts.

The following sentence in the legend of Suppl Fig. 5 did not make sense to me: '2D genomics positions can pairs of peaks of protein of interest from a ChIP-seq experiEment.'(with a typo). Also, the last part of this legend does not seem to look as intended by the authors.

The tables in the manuscript are not formatted as typically requested by academic journals, which should be amended.

Reword ChiA-PET into ChIA-PET.

I wonder why the genome sizes of the speices whose genome assembly was culled from DNA Zoo are so small.

response to reviewers

Reviewer #1 (Remarks to the Author):

The authors present an application of computer vision methods to detect specific contact patterns in HiC contact maps. The theme is of high relevance as more and more HiC datasets become available across different species and cell states. Undeniably, so far the study of these contact maps has been quite limited to the features that were initially identified such as TADs and compartments and despite the recognized importance of chromatin loops, it remains difficult to identify them against noise.
The method is particularly directed at the detection of loops but it can be also used to identify other patterns in the contact maps, which could be just as functionally relevant.
Chromosight, their software, appears to be much faster and more accurate (if we consider sensitivity, which I believe is very important) compared to alternative existing methods.

It is mostly based on performing a convolution of a specific template with the contact map represented as an image, and thus is able to quantify the number of sites where the template is matched and the correlation between the template and the specific region of the contact map can be estimated.

The authors showcase their method on synthetic HiC data mimicking budding yeast. They then proceed to test enrichment of loops detected in budding yeast data in cohesin binding peaks but also show that some of the loops they call are different in mutants are lacking cohesin or having altered loop extrusion.

They then apply the method to human lymphoblastoid cell line GM12878 recovering loops, borders and hairpins structures that are consistent with enrichments of the regions in cohesin and CTCF, as expected. Investigating HiC contact maps from many different animals they identify a conserved loop preferred length of 100kb and they also detect loops from 3C seq of Bacillus Subtilis. Most interestingly, they also detect loops on ChIA-PET data from a virus, the existence of which had been proposed based on other types of observations, which also coincide with occupancy of cohesin and CRCT.

The detection of patterns with their tools is completely user-defined, as they provide a point and click mode of their software to identify templates of interest directly from the data. This can also be used to define a template in one species and recover matching features in another, as shown with pericentromeric inter-chromosomal contacts in 2 yeast species.

The documentation is well written, complete and easy to follow, with all data provided to the user. I installed the software and had no issues. I’m looking forward to using it!

Major Comments:
Generally, I found the paper extremely clear and well-written with clear and informative figures.

We thank the reviewer for the overall appreciation of the work.

I believe there are two main points that could be improved (note that I am not an expert in yeast, bacterial or viral genomes):

Since the authors focus on the GM12878 cell line, whose chromatin contacts have been characterised by a variety of techniques, it would be particularly interesting to see if their loop detection (or indeed detection of other patterns such as hairpins) would be similar starting from the different datasets.
Since the method is able to use virtually any chromosome capture technology as input and it is so efficient, I would have liked to see the results using HiC compared to at least some of the other available datasets for these cells (ChIA-PET, promoter Capture HiC, HiChIP, SPRITE).

More specifically I would be curious to know if the scale of these loops that they identify to peak at 100kb is confirmed across techniques, and if there are other (longer-distance) peaks in this distribution, which are hard to identify in human with HiC.

We thank the reviewer for the suggestion. We have now performed and added analyses of contact data generated using the following technologies:

- *In situ* ChIA-PET of CTCF, from Xingwang Li et al., Nature Protocols 2017
- DNA SPRITE, from Sofia A. Quinodoz et al., Cell 2018
- HiChIP of cohesin, from Maxwell R Mumbach et al., Nature Methods 2016
- Micro-C, from Nils Krietenstein et al., Molecular Cell 2020

We show that Chromosight can easily be applied for pattern detection and quantification on data generated using these 3C-derivative techniques. We present a magnification of the STAT1 gene region for each of the techniques in a new Figure 4A.
These analyses provide clear insights on the different techniques and the ability of Chromosight to identify loops in the different datasets, even noisy ones. The results obtained are largely in agreement between the different datasets, with noise and read coverage being two limiting parameters independent of Chromosight. Micro-C is the approach resulting in the most loops, mostly at short distances thanks to the increase in resolution (Figure 4B).

Chromosight's quantification mode was applied to measure the loop signal on the pairs of cohesin peaks in the different datasets (Figure 4C). Interestingly, a maximum loop signal of ~ 120 kb is found in the different protocols. At longer distances, a secondary peak around 250 kb appears, which would correspond to twice the first peak. These signals would be interesting to compare with physical simulations, and may reflect either a regular underlying disposition of loops or a specific structuration of the chromosome. However, more investigations are needed to elucidate their nature and we decided not to speculate in the manuscript about them.
We added a new subsection named “Application to different proximity ligation protocols” in the Results section to describe these analyses.

I think a little bit more background information should be given considering the wide readership of the journal. Especially a few more sentences about the **differences of chromosome structures across species** would be welcome.

The Introduction has been rewritten to include more details about the shared and different structures found in these different species. The rationale of why we used a specific subset of species is also now better explained.

The detection of **hairpins** and **bow shape** patterns is very interesting but I think the authors should give a few more details about the characteristics of these loops. I found the figures in supplementary helpful but I think a lengthier discussion of the concept could clarify these structures better and highlighted their importance. Also **cohesin injection** should be defined and discussed in the context of recent literature on loop extrusion. This could probably fit in the **introduction**, so the reader has the necessary background to understand the result, or in the **discussion**.

We agree with the referee that this point was not sufficiently explained in the main text. We have expanded the first paragraph of the introduction to better explain what we mean by “hairpin” or “bow shape”, and how they can be formed through mechanisms such as loop extrusion. The kernels are also better described and illustrated in figure 1. We also now included recent references on this topic (for instance on asymmetric loop extrusion) in the introduction. We nevertheless tried to limit the discussion/presentation of the molecular mechanisms responsible for the formation of the loops or other patterns, though, as this is a very actively studied and debated field: instead we refer the reader to recent reviews.

In addition, cohesin injection was actually pretty much a neologism that we removed, as the field does not need extra jargon… Instead we now mention and present “cohesin loading”, which is the standard terminology.

In line 108, some more words should be spent on the possibility that the loops identified corresponds to interacting replichores.

We have clarified this point in the results by adding the following sentence:

> *“Several of these large loops may correspond to the bridging of replichores at positions symmetric with respect to the origin of replication (**Supplementary Fig. 10**). This is in agreement with Marbouty et al. (2015) which showed how SMC condensin SMC-ScpAB complexes loaded at sites adjacent to the origin of replication of the chromosome tether the left and right chromosome arms together while traveling from the origin to the terminus.”*

Maybe it would be good to run some comparison with other methods on a single chromosome of human data even though they are computationally intensive?

A comparison of chromosight with different methods (Hiccups, Hicexplorer and Cooltools) on the human genome is included in the revision. We compared the number of detected loops and their intersection between the different programs, as well as the overlap with CTCF peaks. We present the results in Supplementary Figure 3 and discuss them in the following paragraph

"To get a sense of the differences between the softwares when applied to experimental human contact maps, we compared them with default parameters on Hi-C data generated from GM12878 cell lines (Rao et al., 2014). Compared to Chromosight, we first noticed that other programs missed multiple loops which were clearly visible on the maps (e.g. ***Supplementary Fig. 3a****). For instance, Chromosight found 85% of the loops detected by Cooltools, the software with the highest precision in our benchmark, while overall identifying a much larger number of loops (37,955 vs. 6,264, respectively) (Supplementary Fig. 3c). We then measured the proportion of loops with both anchors overlapping CTCF peaks identified from ChIP-seq (Karolchik, 2004). Almost all (~ 95%) loops detected by Hiccups and Cooltools, the most conservative programs, co-localize with CTCF enriched sites, compared to ~64% for the loops detected by Chromosight and Hicexplorer (****Supplementary Fig. 3b****). Chromosight indeed detects multiple weaker loops, that are nevertheless visible on the maps, and still arranged in grid-like patterns, but with only one anchor falling into a well-defined CTCF enriched site. Some of these weaker loops' anchors may be less enriched in CTCF, which would cause ChIP-seq peak calling algorithms to discard them because of parameters such as intensity thresholds, or minimum inter-peak distances. This means that more sensitive loop callers could result in lower CTCF peak overlap, not because of inaccurate detection, but rather because of the CTCF peaks cutoffs. Alternatively, we can also not exclude that a portion of these less intense loops is linked to different protein complexes or mechanisms."*

Minior issues:
Typos:
Line 80 analysis confirms
Line 81 specific moments
Line 137 which contains

We have corrected these typos.

Vera Pancaldi

Reviewer #2 (Remarks to the Author):

This manuscript introduces the authors' original computation program Chromosight for detecting chromatin structures with computer vision from omics data marking chromatin contacts, such as Hi-C data. While I am convinced by the value and power of the program, I find the manuscript attractive to only a specific range of readers and suitable to a specialized or methodological journal.

Hi-C and related protocols are nowadays a rather streamlined and accessible technology with successful application in various subfields of genomics or genetics. Therefore, tools that facilitate the analysis of such technologies are relevant for a wide range of researchers. Pattern detection methods in Hi-C data have been the focus of recent articles in non-specialized journals (Wang et al., 2017, Nucleic Acid Research; Zufferey et al., 2018, Genome Biology; Ben Zouari et al., 2019, Genome Biology), including Nature Communications (Li et al., 2018; Salameh et al., 2020). In addition, the application of

computer vision on large/various datasets is increasingly popular across fields (for instance, Moen et al., 2019, Nature Methods).

Please supply concrete size information in this part in the Introduction -'bacteria where the sizes of the structures are tiny compared to mammalian genomes'. How tiny are they? Overall, if the authors intend to publish this manuscript in a non-specialized journal, it is strongly recommended to include more biological and concrete information (e.g., SMC protein) in the Introduction as basic introductory items to later support their findings.

We thank the reviewer for the recommendation, which is in line with reviewer 1. We agree that more contextualization of the different species investigated should be provided both in the text and through figures. We have rewritten the introduction, specifying the sizes of the different genomic structures and detailing the biological relevance and background questions at stake.

It is abrupt that in the middle of Introduction, Suppl Fig. 3a is cited without any indication that they performed an analysis to produce these data. As a solution, I would suggest **not to cite any item in Supplementary Material in the Introduction**.

The new introduction does not cite any supplementary material.

The Abstract is not structured to support their conclusion. Especially, these sentences lack the method of comparison and validation. - 'Chromosight has greater sensitivity than existing methods, while being faster and applicable to any type of genomes, including bacteria, viruses, yeasts and mammals. Our results confirm the universal role of Structural Maintenance of Chromosomes proteins (SMC) in the formation of loops at the chromosome level.'

We have modified the first sentence to make it more specific :

> *"Chromosight has greater sensitivity than existing methods on synthetic simulated data, while being faster and applicable to any type of genomes, including bacteria, viruses, yeasts and mammals. "*

Also, the content of the second sentence above is not worth including in the Abstract because it is not their original finding. If insisting on including it, the authors should qualify themselves to include this information just as a method for validating the superiority of their program but not more than that.

We agree and have removed this sentence. Instead, we now highlight the fact that, contrary to methods that aim at characterizing contact patterns using machine learning approaches, our program does not need any training data set and is readily applicable to the genome of any species. We think this is an important point to outline.

Code Availability section provides the link to the GitHub page for Chromosight, but does not include other items to reproduce the benchmarking, etc. presented in the manuscript.

This is an important point. We opted for the creation of a specific github repository allowing to make accessible the codes developed for the analysis as well as to provide some of the intermediate data.

Python scripts and command lines are available on Github:
https://github.com/koszullab/chromosight_analyses_scripts

while intermediate data are available on Zenodo:
https://doi.org/10.5281/zenodo.3742094

Labeling of the horizontal axis for Figure 1b, c, and d is very confusing.

The labels have been adjusted and we added some space between panels to avoid confusion.

Some parts of the texts in the Figures are not consistent in terms of italic/non-italic and capital/lowercases choice.
The abbreviation of the genus names is also inconsistent in some parts.

We have reworked the formatting and labelling to be clearer and more consistent. We now use the complete genus name on the first occurence of each species and only the first letter on subsequent uses.

The following sentence in the legend of Suppl Fig. 5 did not make sense to me: ‘2D genomics positions can pairs of peaks of protein of interest from a ChIP-seq experiEment.’(with a typo). Also, the last part of this legend does not seem to look as intended by the authors.

We have corrected the sentence to: “*Such 2D coordinates can be, for instance, pairs of protein enrichment peaks called from ChIP-seq data*.”

The tables in the manuscript are not formatted as typically requested by academic journals, which should be amended.

We have improved the formatting of tables in PDF and we also provide the tables in a standard text format (.csv) to make their access more convenient.

Reword ChiA-PET into ChIA-PET.

Corrected

I wonder why the genome sizes of the species whose genome assembly was culled from DNA Zoo are so small.

This is a good point raised by the reviewer. It is important to stress that these chromosome-level assemblies and corresponding Hi-C datasets correspond to scaffolds released ‘as-is’ by the repository managing team with a disclaimer that they are a work in progress. They

typically haven't undergone the usual and thorough vetting process of polishing and annotating for general use by the community, because such a process tends to be long and expensive. As such, the reported genome sizes reflected the sum of a subset of scaffolds used to generate the genome-wide contact maps, and not the full assembly. Clearly most of them are broadly underestimated, as smaller scaffolds have been filtered out. Given this is a bit fastidious to explain and not relevant with respect to the efficiency of the program to call loops, we have removed any reference to the genome sizes. In addition, we cannot control for the fact that adjacent contigs will induce "boundary" like signals in the contact map. We have therefore removed the border detection from the supplementary figure. We also removed the hairpin calling given the uncertainties associated with the incompleteness of the genomes.

Nevertheless, loops are visible along these scaffolds that can still be used for genome-wide pattern detection at the scale we work at (Figure S9).
The objectives behind analyzing genomes from DNAZoo are therefore to demonstrate Chromosight's versatility and efficiency on a diverse range of organisms, and to investigate whether the 'loop spectrum' (that is, the distribution of loop sizes) appears not to budge much from a range of 100-150 kb for vertebrates. We hope the reviewer will agree this is an interesting point as well.

We now include a disclosure in the manuscript regarding the nature of the scaffolds used in this analysis.

Reviewer #1 (Remarks to the Author):

I thank the authors for having addressed all my comments. I believe the manuscript is much more complete now, the introduction is much better also for a non-specialist reader and I support its publication.
Two minor typos that I have spotted:
Line 128 bind
Line 133 relevance
